# H3K14 lactylation exacerbates neuronal ferroptosis by inhibiting calcium efflux following intracerebral hemorrhagic stroke

**DOI:** 10.1038/s41419-025-07874-9

**Published:** 2025-07-23

**Authors:** Tingting Sun, Jian-Nan Zhang, Ting Lan, Lei Shi, Liye Hu, Liyan Yan, Chao Wei, Lisha Hei, Weihua Wu, Zhaoli Luo, Meng Liu, Xingmei Ren, Yamei Wang, Yabin Lu, Peipei Wang, Chenguang Zhang, Liheng Bian, Xingquan Zhao, Fei Yang, Qian Li

**Affiliations:** 1 Department of Biochemistry and Molecular Biology, School of Basic Medical Sciences, Capital Medical University, Beijing, China; 2 Department of Neurobiology, School of Basic Medical Sciences, Capital Medical University, Beijing, China; 3 School of Criminal Investigation, People’s Public Security University of China, Beijing, China; 4 Department of Neurology, Beijing Tiantan Hospital, Capital Medical University, Beijing, China; 5 Laboratory for Clinical Medicine, Capital Medical University, Beijing, China

**Keywords:** Stroke, Diseases of the nervous system

## Abstract

Inhibiting neuronal ferroptosis is essential for mitigating neural damage and enhancing recovery in central nervous system (CNS) disorders, including intracerebral hemorrhagic stroke (ICH). Lactate accumulation correlates with ICH severity, yet the role of lactate-derived histone lactylation, a novel epigenetic modification, in ferroptosis and its mechanisms is not fully understood. In this study, we aimed to investigate the role of histone lactylation on neuronal ferroptosis in ICH models, both in vitro and in vivo. We discovered elevated lactate and histone lactylation post-ICH in mice, with a significant increase in H3K14la during the early stages of ferroptosis in hemin-challenged primary cortical neurons. Pharmacological or genetic inhibition of H3K14la by targeting lactate dehydrogenase (LDH) enzyme activity effectively suppressed neuronal ferroptosis. We further identified p300/CBP and class I histone deacetylases (HDACs) as the key modifiers of H3K14la in this process. Through chromatin immunoprecipitation-sequencing and RNA-sequencing (RNA-seq) in hemin-treated neurons, we pinpointed the Ca^2+^-ATPase PMCA2 encoding gene as a direct downstream target of H3K14la. H3K14la/PMCA2 promoted ferroptosis by elevating intracellular calcium levels. In line with our in vitro findings, inhibiting H3K14la/PMCA2 reduced neuronal degeneration and improved functional outcomes in an ICH mouse model induced by intracranial injection of collagenase into the striatum. Taken together, our findings elucidate the role of histone lactylation and PMCA2 in neuronal ferroptosis and implicate that targeting histone lactylation could be a promising therapeutic strategy for ICH and related CNS diseases.

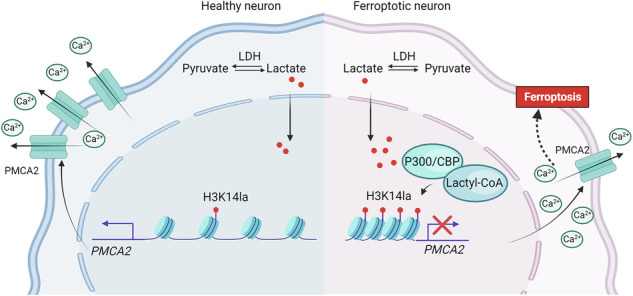

## Highlights

- Histone lactylation, especially H3K14la, contributes to neuronal ferroptosis in the early stage after ICH.
- Ca^2+^-ATPase PMCA2 encoding gene is the downstream target of H3K14la.
- H3K14la/PMCA2 promotes neuronal ferroptosis by increasing the intracellular levels of calcium.

## Introduction

Ferroptosis, first characterized in cancer cells in 2012, is a unique form of cell death driven by iron-dependent phospholipid peroxidation, which is apart from other forms of cell death pathways, such as apoptosis, necroptosis, pyroptosis [1, 2]. It can be inhibited by factors that either block lipid peroxidation directly or deplete iron. Subsequently, glutathione peroxidase 4, Ferroptosis suppressor protein 1, dihydroorotate dehydrogenase, and 6(R)-l-erythro-5,6,7,8-tetrahydrobiopterin were recognized as endogenous inhibitory mechanisms of ferroptosis [3–5]. It is also regulated by diverse pathways such as lipid peroxidation, glutathione depletion, iron metabolism, mitochondrial activity, and the metabolism of amino acids, lipids and sugars [2]. An increase of cytosolic Ca^2+^ is a hallmark of ferroptosis before the complete bursting of the cell [6] and the plasma membrane Ca^2+^ ATPases (PMCA) are responsible for pumping Ca^2+^ [7]. Our recent research, along with others, has revealed that ferroptosis plays a role in various central nervous system (CNS) disorders, including intracerebral hemorrhagic stroke (ICH), ischemic stroke, traumatic brain injury, multiple sclerosis, and Parkinson’s disease [8–13]. Importantly, all these findings indicate that protecting neurons against ferroptosis significantly improves outcomes in animal models of these conditions.

ICH is the most fatal stroke subtype, characterized by the highest rates of mortality and morbidity [14]. Despite its severity, effective treatments are lacking in the clinic [15]. ICH is diagnosed when bleeding occurs within the brain’s parenchyma often due to hypertension [16]. Following ICH, the formation and expansion of a hematoma led to primary brain damage, while hemoglobin, hemin, and iron released from ruptured red blood cells are mainly responsible for secondary brain damage and neurological deficits [17]. Iron overload is found in the perihematomal brain tissues and especially in neurons in the hemorrhagic brain [12, 18, 19]. The presence of excessive Fe^2+^ in neurons triggers the Fenton reaction, leading to an increase in lipid reactive oxygen species (ROS) levels and the initiation of ferroptosis [13, 20]. Emerging evidence has shown that inhibiting ferroptosis can have beneficial effects in both in vivo and in vitro models of ICH [12, 20]. Therefore, it is essential to further understand the mechanisms of neuronal ferroptosis following ICH and to develop targeted interventions to suppress ferroptosis, which could lead to the discovery of effective therapeutic strategies for ICH.

Historically regarded as a byproduct of glucose metabolism, lactate has recently been an important signaling molecule in the brain [21]. Lactate accumulation in the patient serum and mouse brains post-ICH [22, 23], and a recent study has linked an initial increase in lactate levels to poor outcomes in ICH patients [22]. Yet, the precise role of lactate in ICH remains to be fully understood. Most recently, lactate has been implicated in histone lactylation, an epigenetic modification that can directly influence gene transcription [24, 25]. Lactyl coenzyme A provides the donor [26] and thus lactyl group is transferred to lysine residues on histones by histone acetyltransferases such as p300/CBP [27]. In vitro experiments have shown that class I histone deacetylases (HDAC1-3) can erase the Lactyl group [28]. This type of modification has been associated with various pathological conditions, including neuronal excitability, infection, cancer, and Alzheimer’s disease [29–33]. Emerging evidence underscores the importance of epigenetic regulation in the pathology of neuronal ferroptosis [5, 34]. Thus, investigating the potential roles of lactate-derived histone lactylation in the progression of neuronal ferroptosis after ICH could reveal novel therapeutic strategies for the treatment of ICH.

## Results

### The levels of H3K14la were increased in ferroptotic neurons after ICH

**Fig. 1:**
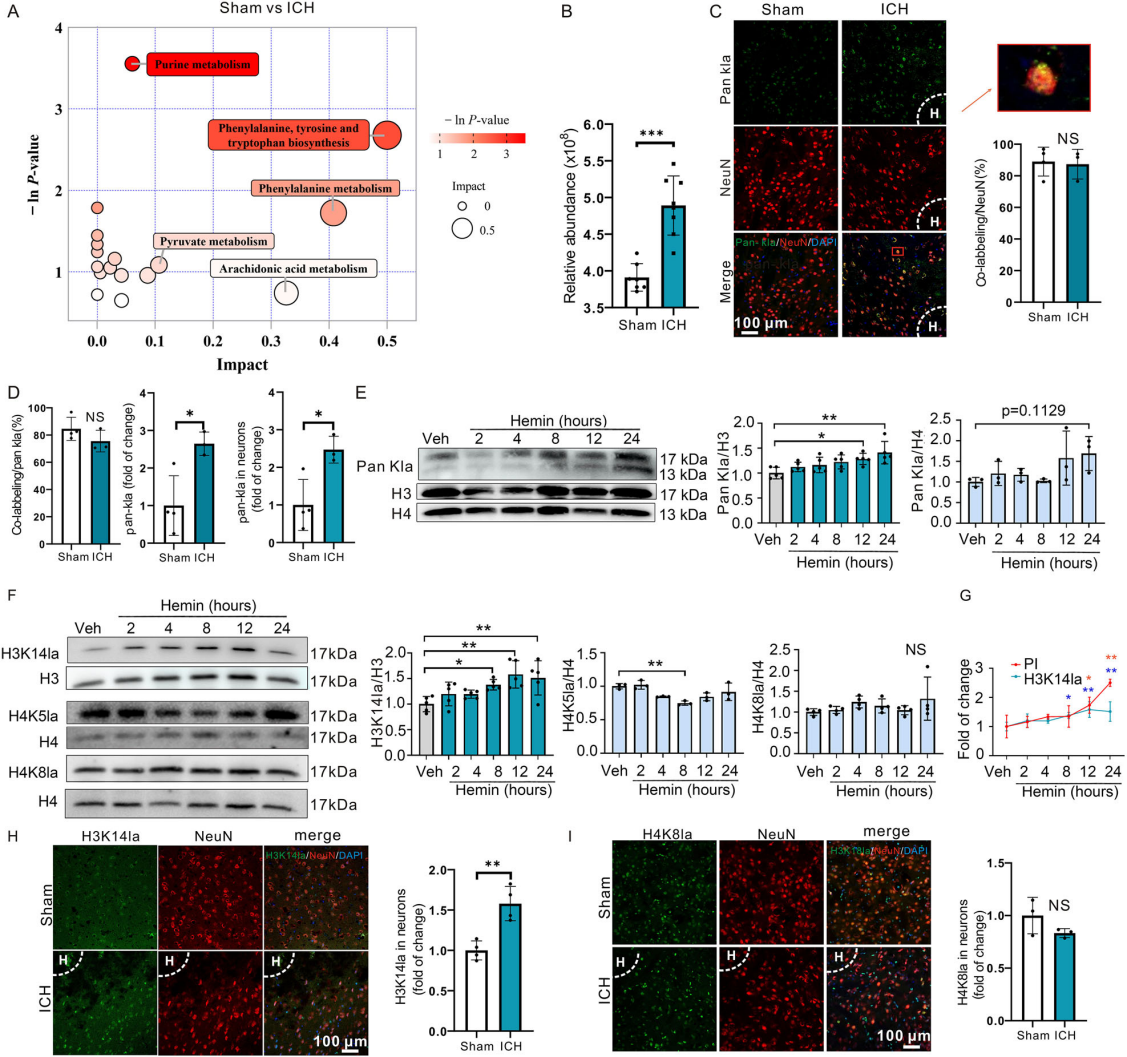
The levels of H3K14la were increased in ferroptotic neurons after ICH. The untargeted metabolomics analysis was performed to characterize the metabolic changes in sham and ICH mice. The biology diagram shows the metabolic pathway in sham and ICH mice (**A**). The lactic acid content between the two groups is shown (**B**). (sham group: n = 7 mice; ICH group: n = 8 mice). **C**, **D** Immunostaining of pan Kla and NeuN was performed after ICH. “H” indicates hematoma. The representative images and quantifications are shown. (sham group: n = 4 mice; ICH group: n = 3 mice) **E**, **F** The levels of pan Kla, H3K14la, H4K5la and H4K8la were examined by Western blotting. (pan Kla: n = 5 cultures; H3K14la: n = 5 cultures; H4K5la: n = 3 cultures; H4K8la: n = 4 cultures). **G** The levels of H3K14la and PI staining in PCNs at different time points upon hemin or Veh treatment were compared. **H**, **I** Immunostaining of H3K14la, H4K8la and NeuN was performed after ICH. “H” indicates hematoma. (H3K14la: n = 4 mice; H4K8la: n = 3 mice) The representative images and quantifications are shown. Results are shown as (**B**–**F** and **H**–**I**) scatter plots (Mean ± SD) or (**G**) line graphs (Mean ± SD). Unpaired two-tailed Student’s *t* test (**B**–**D** and **H**–**I**) or one-way ANOVA followed by Dunnett’s multiple comparisons tests (**E**–**G**) was used. *p < 0.05, **p < 0.01, ***p < 0.001vs Veh; NS, not significant.

We performed an untargeted metabolomic analysis using liquid chromatography-mass spectrometry (LC-MS) on ipsilateral mouse brains after intracranial injection of collagenase into the striatum [35]. Previous evidence has shown that lactate accumulation was correlated to poor outcomes in ICH patients as well as to neuronal ferroptosis in mice [22, 35]. Given that the pyruvate pathway is closely linked to ferroptosis [36], we re-analyzed this data and focused on the pyruvate pathway, which is related to lactate metabolism (Fig. 1A). Our analysis also revealed a significant increase in lactate production in ICH mice compared to the sham-operated group (Fig. 1B). To explore the presence of histone lactylation in neurons at the perihematomal region, we performed co-immunostaining with pan Kla (a marker for protein lactylation) and NeuN (a neuronal marker) antibodies. The results indicated that the pan Kla signal predominantly colocalized with DAPI, and the majority of pan-Kla^+^ cells were also NeuN^+^ neurons (Fig. 1C, D). Notably, the levels of pan Kla in neurons were dramatically increased in the perihematomal region of mice at 24 h after ICH compared to the sham-operated group (Fig. 1C, D). Given that our previous study showed that ferroptosis pathway-related metabolites had a significant increase in ICH mice [35], the above data suggests that histone lactylation may play a role in neuronal ferroptosis following ICH.

To investigate the role of histone lactylation in neuronal ferroptosis after ICH, we cultured PCNs from mouse pups and used hemin to mimic hemorrhagic damage in vitro, as described in previous studies [37]. Immunostaining revealed the purity of PCNs on day in vitro (DIV) 7 without glia cells (Fig. S1A, B). Hemin induced neuronal death in a dose-dependent (Fig. S1C, D) and time-dependent manner compared with the vehicle (Veh) group (Fig. S1E, F). The accumulation of lipid ROS and cell death induced by hemin was effectively mitigated by ferroptosis inhibitors Ferrostain-1 (Fer-1) and Deferoxamine (DFO) (Fig. S1G-J). Furthermore, hemin also increased the overexpression of *Ptgs2*, a hallmark of ferroptosis (Fig. S1K). All these findings confirm the successful establishment of an in vitro model of hemin-induced neuronal ferroptosis.

We then examined the changes in pan Kla levels in hemin-treated neurons. In line with our in vivo observations, the expression of Kla on histone 3 significantly increased post-hemin treatment and remained elevated up to 24 h, whereas the expression of Kla on histone 4 showed no such increase (Fig. 1E). Further analysis of different lysine residues on histone 3 revealed a notable increase in H3K14la levels in neurons after hemin treatment, which persisted for up to 24 h (Fig. 1F). Conversely, the expression of H4K5la decreased and no notable changes were observed in H4K8la over time (Fig. 1F). Additionally, we assessed the fold changes in both cell death and H3K14la levels at various time points following hemin treatment. Our findings indicate that hemin treatment began to elevate H3K14la levels at 8 h and triggered neuronal ferroptosis by 12 h (Fig. 1G). This suggests that histone lactylation, particularly H3K14la, may be involved in ferroptosis. These results were corroborated by in vivo experiments, which showed increased levels of H3K14la, but not H4K8la, in the perihematomal region of mice at 24 h after ICH (Fig. 1H, I). However, most of the well-characterized epigenetic mechanism was not involved in neural ferroptosis (Fig. S2).

Collectively, our findings indicate that the levels of H3K14la were specifically elevated in neurons undergoing ferroptosis following ICH.

### Inhibition of H3K14la suppressed hemin-induced neuronal ferroptosis in vitro

**Fig. 2:**
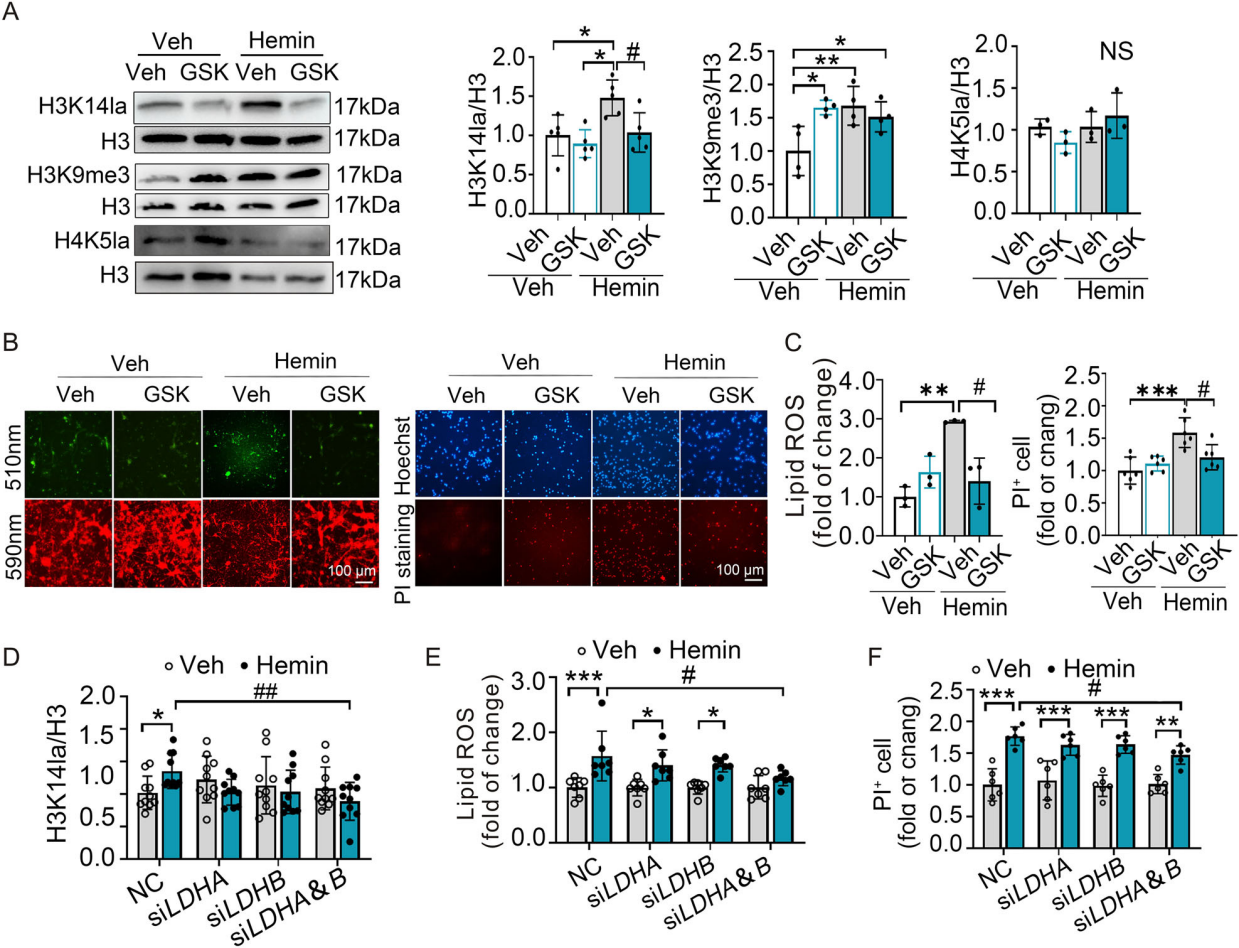
Inhibition of LDH activity suppressed hemin-induced neuronal ferroptosis in vitro. **A** After the indicated treatment of PCNs for 12 h, the levels of H3K14la, H3K9me3, and H4K5la were examined using Western blotting. H3 serves as a loading control. (H3K14la: n = 5 cultures; H3K9me3: n = 4 cultures; H4K5la: n = 3 cultures). BODIPY 581/591 C11 reagent was used to detect the changes of intracellular lipid ROS after 24 h treatment with 50 µM hemin and 50 µM GSK, and the cell death was detected by PI and Hoechst staining (**B**). The quantifications for cell death and the intracellular lipid ROS are shown (**C**). (lipid ROS: n = 3 cultures; PI: n = 6 cultures). **D** After LDHA and LDHB were silenced by siRNA, the expression of H3K14la was detected by Western blotting at 12 h after 50 µM hemin treatment in PCNs. n = 10 cultures. **E** After transfection with siRNA of LDHA and LDHB, the lipid ROS of 50 µM hemin-treated PCNs were detected at 24 h by BODIPY 581/ 591 C11 reagent. n = 7 cultures. **F** After transfection with siRNA of LDHA and LDHB, the cell viability of hemin-treated PCNs was detected at 24 h by PI and Hoechst staining. n = 6 cultures. Results are shown as scatter plots (Mean ± SD). One-way ANOVA followed by Tukey’s multiple comparison tests (**A**, **C**), or two-way ANOVA followed by Sidak (**D**–**F**) multiple comparison tests was used. *p < 0.05, **p < 0.01, ***p < 0.001 vs Veh; #*p* < 0.05, ##*p* < 0.01 vs Hemin; NS not significant.

To elucidate the role of H3K14la in neuronal ferroptosis, PCNs were co-treated with hemin and the lactate dehydrogenase (LDH) inhibitor GSK [38], which inhibits lactate production and, consequently, reduces Kla levels. As anticipated, GSK treatment led to a decrease in H3K14la accumulation in neurons exposed to hemin, without affecting the levels of H3K9me3, a histone modification that significantly changes after hemin treatment or H4K5la between the hemin + Veh and hemin + GSK groups (Fig. 2A). Importantly, the accumulation of lipid ROS and cell death induced by hemin were substantially reduced with the addition of GSK (Fig. 2B, C). To validate these results, neurons were co-treated with hemin and another LDH inhibitor, oxamate [39], which also significantly mitigated the hemin-induced increase in lipid ROS and cell death (Figs. S3A, B). Acknowledging the potential off-target effects of GSK and oxamate [39, 40], we knocked down the expression of LDH-encoding genes *LDHA* and *LDHB* using specific siRNAs (Fig. S3C-E). Silencing both *LDHA* and *LDHB* markedly suppressed the hemin-induced increase in H3K14la levels (Fig. 2D and S3F). The double knockdown of *LDHA* and *LDHB* also consistently reduced lipid ROS accumulation (Fig. 2E and S3G) and cell death (Fig. 2F and S3H) in neurons treated with hemin. The above data demonstrate that modulating LDH activity to inhibit H3K14la accumulation suppresses hemin-induced neuronal ferroptosis.

**Fig. 3:**
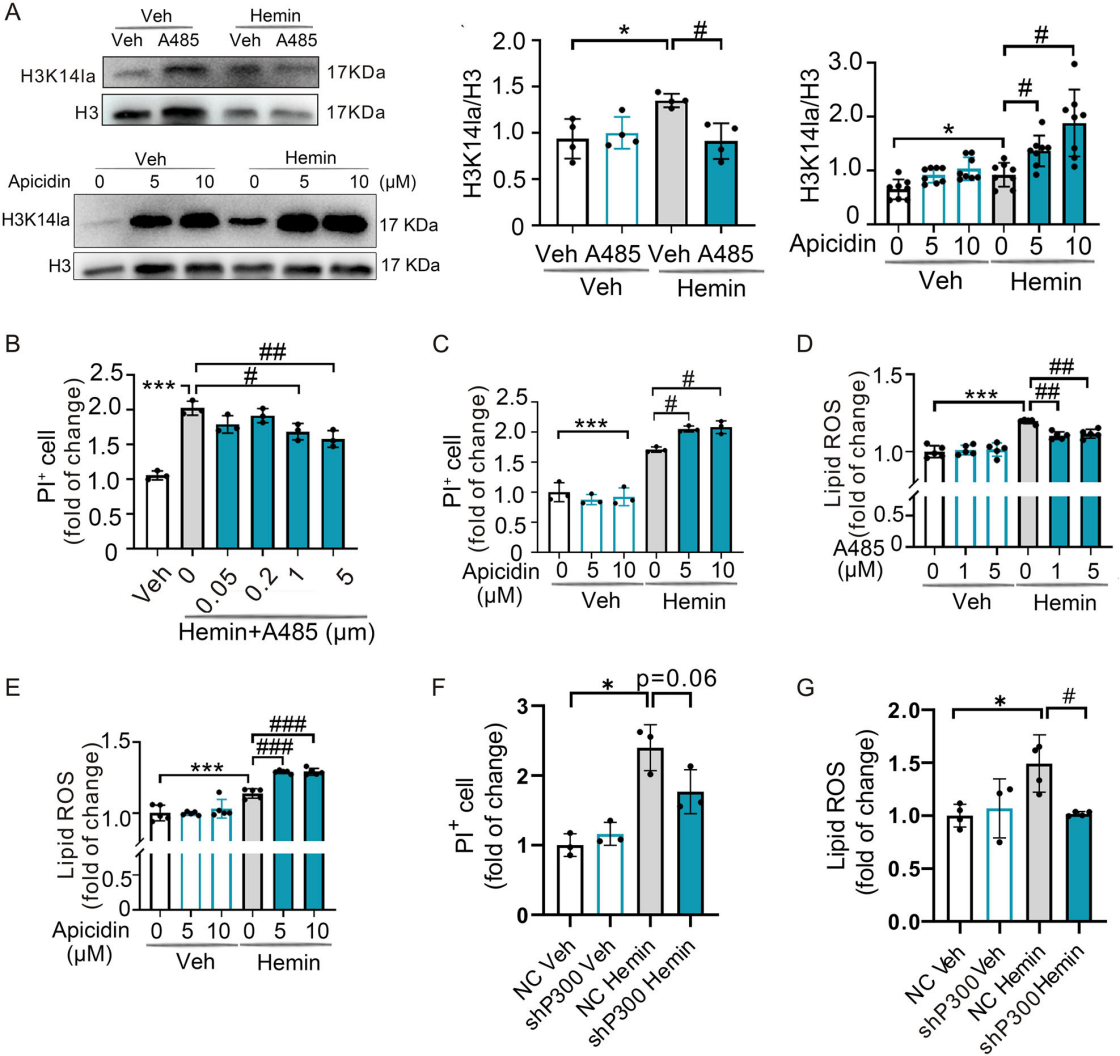
P300/CBP and class I HDAC regulated neuronal ferroptosis by affecting H3K14la levels in vitro. **A** After hemin treatment of PCNs at 12 h, the expression of H3K14la was detected after 50 µM hemin treatment and combined administration with A485 or Apicidin (left side: n = 4 cultures; right side: n = 8 cultures). **B**, **C** The changes in cell death after 50 µM hemin and A485 or Apicidin combined treatment later 24 h were detected by PI and Hoechst staining (**B**: n = 3 cultures; **C**: n = 3 cultures). **D**, **E** BODIPY 581/ 591 C11 reagent was used to detect the changes of intracellular lipid ROS later at 24 h after treatment with 50 µM hemin and A485 or Apicidin. n = 5 cultures. **F** The cell death of PCNs transduced with the AAV-hSyn-EGFP-NC (NC) or AAV-hSyn-EGFP-shP300 (shP300) at 24 h after hemin treatment was detected by PI and Hoechst staining. n = 3 cultures. **G** BODIPY 581/591 C11 reagent was used to detect the changes of intracellular lipid ROS in PCNs transduced with AAV virus NC or shp300 at 24 h after hemin treatment. Results are shown as scatter plots (Mean ± SD). n = 3-4 cultures. One-way ANOVA followed by Tukey’s multiple comparisons tests was used. *p < 0.05, ***p < 0.001 vs Veh; #*p* < 0.05, ##*p* < 0.01, ###*p* < 0.001 vs Hemin.

We subsequently examined the impact of p300 (a histone lysine lactylase) and class I HDACs (histone lysine delactylases) on neuronal ferroptosis. Our findings revealed that A485, a recognized inhibitor of P300/CBP, markedly reduced the H3K14la levels in neurons exposed to hemin, while Apicidin, which specifically inhibits HDAC2 and HDAC3 activity, dramatically increased H3K14la levels in these neurons (Fig. 3A). Moreover, A485 treatment effectively inhibited cell death (Fig. 3B and S4A) and the accumulation of lipoid ROS (Fig. 3D and S4C) triggered by hemin, while Apicidin aggravated hemin-induced cell death (Fig. 3C and S4B) and lipoid ROS accumulation (Fig. 3E and S4D). Additionally, the knockdown of *p300* using AAV-hSyn-EGFP-shP300 had a trend to attenuate cell death (Fig. 3F and S4E) following hemin treatment and also inhibited lipid ROS accumulation induced by hemin (Fig. 3G and S4F) following hemin treatment. Thus, our data indicate that P300/CBP and class I HDACs play a regulatory role in hemin-induced neuronal ferroptosis by modulating H3K14la levels.

Collectively, the above data indicate that inhibition of H3K14la suppressed neuronal ferroptosis induced by hemin in vitro.

### H3K14la orchestrates neuronal ferroptosis by modulating the transcription of PMCA2 in vitro

**Fig. 4:**
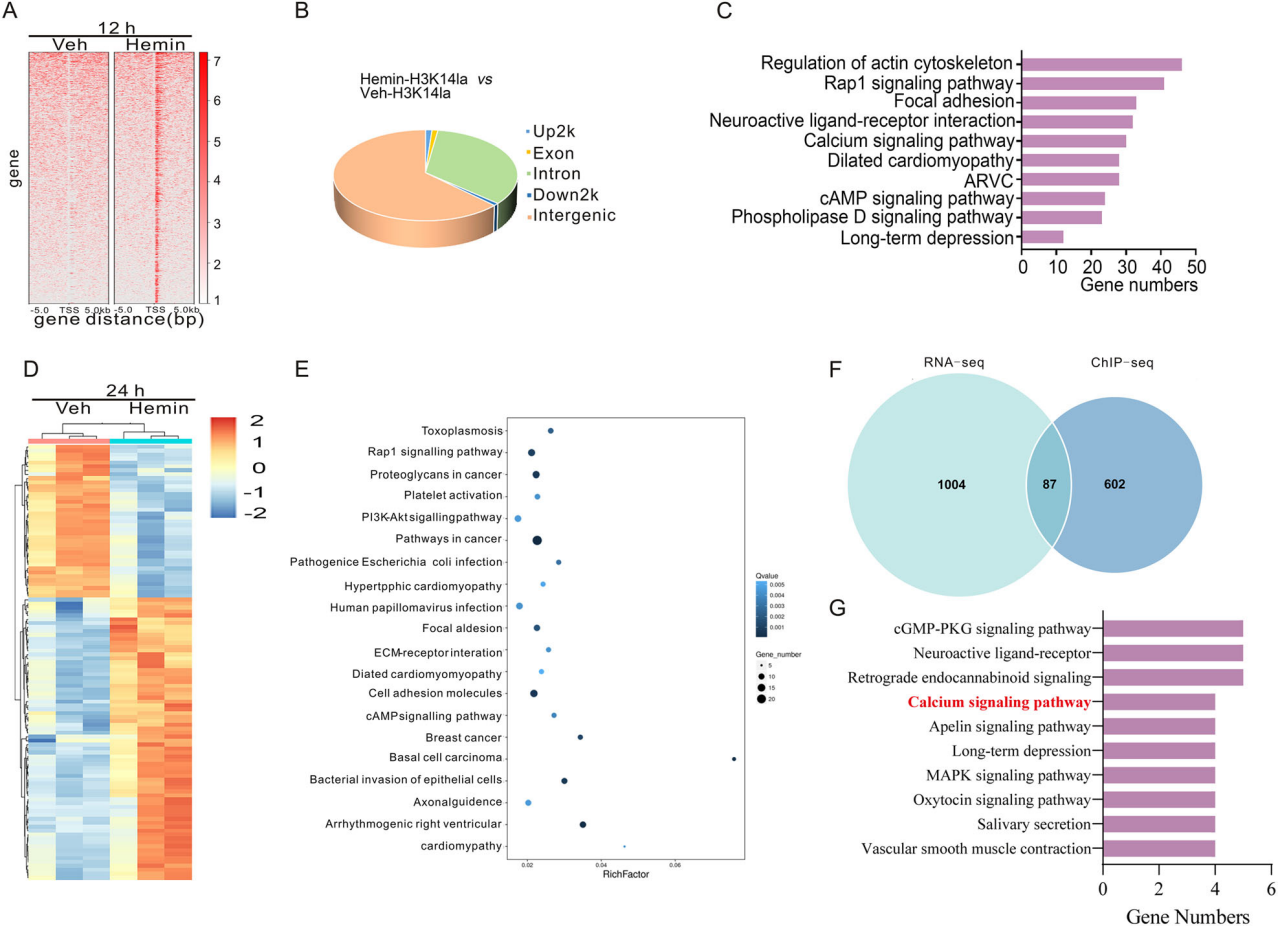
Identification of the potential target genes of H3K14la regulating ferroptosis after hemin treatment in vitro. **A** The binding density of H3K14la was visualized by deepTools: the heatmap presents different H3K14la binding peaks in N2A cells between Veh and hemin groups. **B** ChIP-Seq detected the genomic distribution of the H3K14la in the Veh and hemin-treated N2A cells at 12 h. **C** KEGG analyses of annotated targets of H3K14la modification in hemin treated group based on ChIP-Seq data. **D** The RNA-Seq results from 24 h in neurons treated with Hemin. **E** KEGG analysis of annotated targets based on the RNA-Seq data. **F** Venn diagram of RNA-Seq and ChIP-Seq. **G** KEGG analysis of annotated targets of H3K14la modification in hemin treated group based on ChIP-Seq data with RNA-Seq data.

To reveal the underlying molecular mechanisms of how H3K14la regulated neuronal ferroptosis, we performed chromatin immunoprecipitation sequencing (ChIP-seq) using anti-H3K14la antibodies to identify potential target genes of H3K14la. We observed an enrichment of H3K14la peaks in the hemin-treated cells compared to Veh-treated cells (Fig. 4A). The genomic distribution analysis revealed a preference for H3K14la to bind intergenic and intron regions in hemin-treated cells (Fig. 4B). Kyoto Encyclopedia of Genes and Genomes (KEGG) analysis, classified these distinct H3K14la binding peaks, implicating pathways such as the calcium signaling pathway, cAMP signaling pathway, and Rap1 signaling pathway (Fig. 4C).

By comparing the differential genes from the ChIP-seq results with the FerrDb database, we found overlapping genes *FBXW7*, *ZEB1*, and *TFR2* (Fig. S5A). Out of these three genes, the expression levels of *FBXW7* and *TFR2* were markedly reduced following hemin treatment but not *ZEB1* (Fig. S5B). Given that *TFR2* is primarily expressed in the cerebellum and ICH typically occurs in the striatum [41], we concentrated on the role of *FBXW7* in H3K14la-mediated neuronal ferroptosis. ChIP-qPCR and agarose gel electrophoresis assays confirmed a significant enrichment of H3K14la at the *FBXW7* gene locus (266-592 bp) after hemin treatment (Figs. S5C-D). However, knocking down *FBXW7* expression with siRNA (Fig. S5E) did not affect cell death or lipid ROS accumulation in hemin-induced neuronal ferroptosis (Figs. S5F-H). This suggests that H3K14la regulates neuronal ferroptosis independently of *FBXW7* or other well-characterized ferroptosis-related genes.

**Fig. 5:**
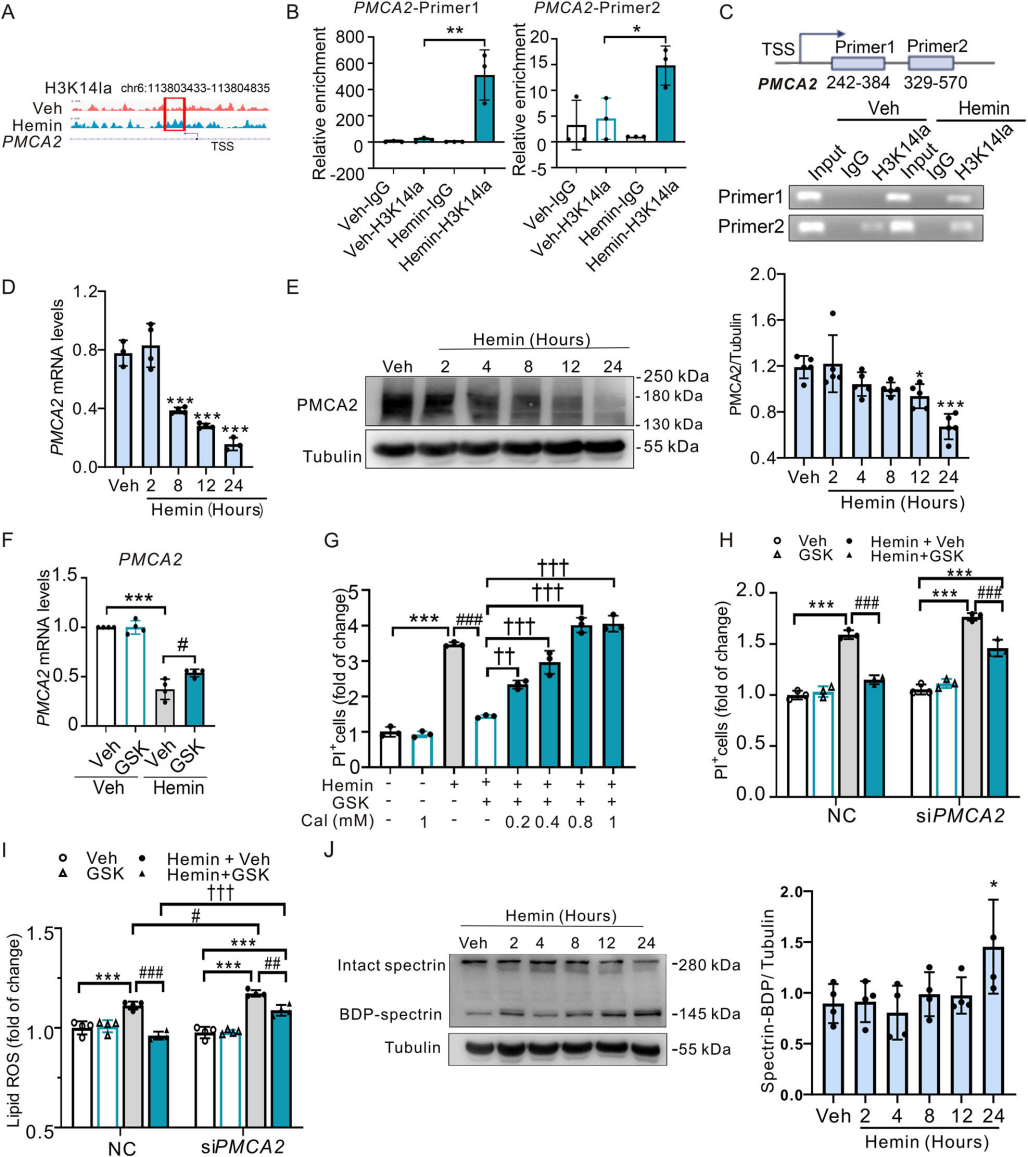
H3K14la/PMCA2 axis promotes ferroptosis by intracellular Ca^2+^ in PCNs after hemin treatment in vitro. **A** The peaks of target genes were analyzed by an integrative genomics viewer. The red and blue respectively indicate the peak regions of H3K14la on the target gene about Veh and hemin group. n = 3 cultures. **B** The enrichment of H3K14la in PMCA2 was detected by ChIP-PCR in Veh and hemin treatment groups in N2A cells. **C** Design location of primers and results of agarose gel electrophoresis. **D** mRNA levels of *PMCA2* were detected by RT-PCR at indicated time points after 50 µM hemin treatment. n = 3-4 cultures. **E** The expression of PMCA2 was detected at indicated time points after 50 µM hemin treatment. n = 5 cultures. **F** mRNA levels of PMCA2 were detected by RT-PCR after 50 µM hemin with GSK treatment of PCNs at 12 h. GAPDH serves as the internal control. n = 4 cultures. **G** The changes in cell death after 50 µM hemin and Caloxin 2a1(Cal) combined treatment later 24 h were detected by PI and Hoechst staining. n = 3 cultures. **H** After transfection with siRNA of PMCA2, the changes in cell death after 50 µM hemin or/and GSK combined treatment later 24 h were detected by PI and Hoechst staining. n = 3 cultures. **I** After transfection with siRNA of PMCA2, the lipid ROS of 50 µM hemin-treated PCNs with combined treatment of GSK were detected at 24 h by BODIPY 581/ 591 C11 reagent. n = 4 cultures. **J** The expression of spectrin was detected at indicated time points after 50 µM hemin treatment. Tubulin serves as the loading control. n = 4 cultures. Results are shown as scatter plots (Mean ± SD). One-way ANOVA followed by Tukey’s multiple comparisons tests (**B** and **F**–**I**), or Dunnett’s (**D**, **E**, **J**) multiple comparisons tests were used, *p < 0.05, **p < 0.01, ***p < 0.001 *vs* Veh; #*p* < 0.05; ##*p* < 0.01, ###*p* < 0.001 vs Hemin +Veh; ††*p* < 0.01, †††*p* < 0.001 vs Hemin + GSK.

Next, we re-analyzed RNA-seq data on neurons 24 h after treatment with hemin or Veh (GEO, https://www.ncbi.nlm.nih.gov/geo/, GSE189652), coinciding with the peak of cell death (Fig. 4D). The differentially expressed genes were categorized into various pathways using KEGG analysis (Fig. 4E). By aligning ChIP-seq data with RNA-seq data, we identified 87 genes that were both H3K14la-targeted and differentially expressed in hemin-treated cells (Fig. 4F). These genes were enriched in multiple signaling pathways, notably the calcium signaling pathway (Fig. 4G), which has been recently implicated in ferroptosis [42]. Among the identified candidate genes, *PMCA2* is particularly significant as it encodes the plasma membrane Ca^2+^-ATPase 2 (PMCA2), responsible for the transport of Ca^2+^ from the cytoplasm to the extracellular space [7]. The ChIP-seq analysis showed that H3K14la was notably enriched within the gene body of *PMCA2* in cells treated with hemin (Fig. 5A). Further validation through agarose gel electrophoresis and qPCR following the ChIP procedure confirmed the substantial enrichment of H3K14la in the *PMCA2* gene at genes spanning 242-384 bp and 329-570 bp after hemin treatment (Fig. 5B, C). Subsequent analysis confirmed that the expression of *PMCA2* was notably reduced at both mRNA (Fig. 5D) and protein (Fig. 5E) levels following hemin treatment.

To further examine whether H3K14la regulated neuronal ferroptosis through *PMCA2*, we administered a co-treatment of hemin and GSK to the neurons. Our findings indicated that GSK treatment markedly restored the expression levels of *PMCA2*, which had been suppressed by hemin (Fig. 5F). Subsequently, we exposed neurons to Caloxin 2a1, an inhibitor of the PMCA channel known to elevate intracellular calcium levels, in combination with hemin and GSK. Notably, Caloxin 2a1 effectively mitigated the protective effect of GSK on cell death (Fig. 5G). Consistent with these findings, the knockdown of *PMCA2* also negated the protective effects of GSK on cell death and the accumulation of lipid ROS triggered by hemin (Fig. 5H, I). Furthermore, we examined the expression levels of BDP-spectrin, a marker commonly used to measure the proteolytic activity of the calcium-dependent protease calpain [43]. An increase in BDP-spectrin expression was observed after hemin treatment, suggesting that hemin treatment increased intracellular Ca^2+^ levels (Fig. 5J).

Taken together, these results suggest that H3K14la regulates hemin-induced neuronal ferroptosis by targeting *PMCA2*, thereby influencing intracellular Ca^2+^ homeostasis.

### Inhibition of the H3k14la/*PMCA2* axis protects against hemorrhagic brain injury in vivo

**Fig. 6:**
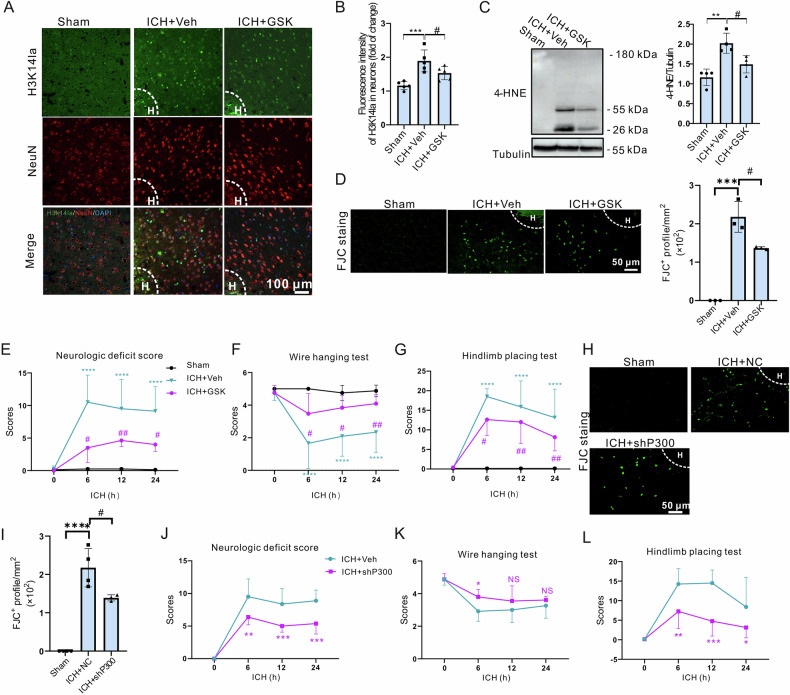
Inhibition of H3K14la improved functional outcomes after ICH in mice. Immunostaining of H3K14la and NeuN was performed at 24 h after ICH. “H” indicates hematoma. The representative images (**A**) and quantifications (**B**) are shown. n = 5 mice. **C** The expression of 4-HNE was measured at 24 h after ICH by western blot analysis. Tubulin serves as the loading control. n = 4 mice. **D** FJC staining was performed at 24 h after ICH. The representative images and quantification are shown. n = 4 mice. **E–G** 1 μl of 500 μM GSK or 10 mM Caloxin 2A1 was injected at 30 min after ICH. Neurological function and motor function were determined at indicated time points after ICH. n = 8 mice. FJC staining was performed at 24 h after ICH. The representative images (**H**) and quantification (**I**) are shown. n = 4 mice. **J–L** AAV virus of NC or shP300 virus was injected at 1 week before ICH and Neurological function and motor function were examined at indicated time points after ICH. Results are shown as scatter plots (Mean ± SD). n = 8 mice. One-way (**B**–**D** and **I**) or two-way (**E**–**G** and **J**–**L**) ANOVA followed by Tukey’s multiple comparisons tests was performed, *p < 0.05, **p < 0.01, ***p < 0.001, ****p < 0.0001vs Sham; #*p* < 0.05 vs ICH + NC/Veh; NS, not significant.

To confirm the role of H3k14la in neuronal protection following ICH in vivo, we administered GSK intracerebroventricularly to mice 30 min after intracranial injection of collagenase into the striatum. Co-immunostaining for NeuN and H3k14la showed that H3K14la levels in neurons were increased in the perihematomal region 24 h post-ICH compared to the sham group (Fig. 6A, B). However, GSK injection effectively inhibited this increase after ICH (Fig. 6A, B). We next assessed the expression levels of 4-hydroxynonenal (4-HNE), an end product of lipid peroxidation and a hallmark of ferroptosis. Our results indicated that 4-HNE levels were elevated at 24 h post-ICH and that GSK treatment led to a marked reduction in 4-HNE expression levels (Fig. 6C). In addition to this, the profile of Fluoro-Jade C (FJC)^+^ degenerating neurons (Fig. 6D) and the neurologic/motor function deficits (Fig. 6E-G) observed in ICH mice were also effectively ameliorated by GSK administration. These results were further confirmed by the knockdown of *p300* in striatal neurons using AAV-hSyn-EGFP-shP300. Knockdown of *p300* not only suppressed the increase of FJC^+^ profiles in the perihematomal region after ICH (Fig. 6H, I) but also improved the acute behavioral outcomes as assessed at 24 h post-ICH (Fig. 6J-L).

**Fig. 7:**
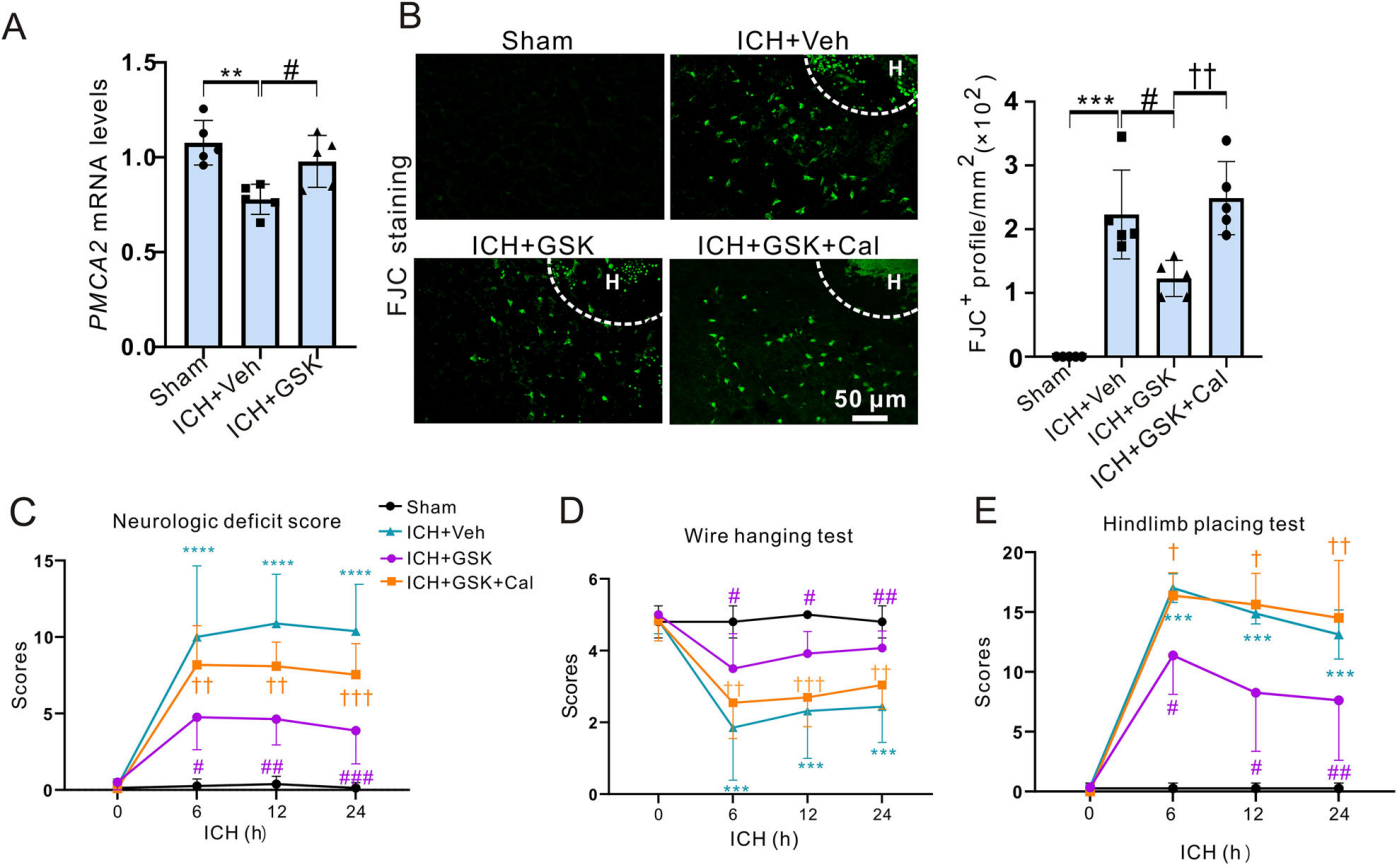
PMCA2 was one downstream target of H3k14la after ICH in mice. **A** mRNA levels of PMCA2 were detected by RT-PCR at 24 h after ICH. GAPDH serves as the internal control. n = 5 mice. **B** FJC staining was performed at 24 h after ICH. The representative images and quantification are shown. n = 5 mice. (**C–E**) 1 μl of 500 μM GSK or 10 mM Caloxin 2A1 was injected at 30 min after ICH. Neurological function and motor function were determined at indicated time points after ICH. n = 8 mice. Results are shown as scatter plots (Mean ± SD). One-way (**A**, **B**) or two-way (**C**-**E**) ANOVA followed by Tukey’s multiple comparisons tests was performed, **p < 0.01, ***p < 0.001, ****p < 0.0001 vs Sham; #*p* < 0.05, ##*p* < 0.01, ###*p* < 0.001vs ICH + Veh; †*p* < 0.05, ††*p* < 0.01, †††*p* < 0.001 *vs* ICH + GSK.

Next, we explored the role of the H3K14la/PMCA2 axis in vivo. As expected, the administration of GSK markedly mitigated the reduction in *PMCA2* mRNA expression observed 24 h after ICH (Fig. 7A). Furthermore, the PMCA channel inhibitor Caloxin 2a1 reversed the neuroprotective effects of GSK on neuronal degeneration (Fig. 7B) and neurologic functions (Fig. 7C-E).

In summary, these findings collectively indicate that the H3K14la/*PMCA2* axis aggravated neuronal death and the acute outcomes after ICH.

## Discussion

In ICH patients, elevated lactate and glucose levels immediately following ICH are associated with an unfavorable prognosis [22]. Interestingly, in an ICH rat model, lactate infusion was found to stimulate angiogenesis and neurogenesis, and to reduce nerve regeneration and apoptosis [23]. In addition, the administration of oxamate, an LDH inhibitor, aggravated the pathological outcomes after ICH in these rats [23]. In our study, we discovered that lowing lactate levels by the addition of the LDH inhibitors GSK/oxamate or by genetically silencing the LDH-encoding gene *LDHA*/*LDHB* in neurons effectively inhibited neuronal ferroptosis as well as improved the outcomes after ICH. These data suggest that the reduction of neuronal lactate may serve as a protective effect against ICH by inhibiting neuronal ferroptosis. These contradictory results indicate that lactate may play different roles in neurons, glial cells, and immune cells after ICH. More importantly, lactate may have distinctive effects on metabolism and gene transcription reprogramming.

Histone lactylation, a recently identified epigenetic modification in macrophages [24], has not yet been explored in the context of neuronal ferroptosis and ICH. Different lactylation sites have been identified on core histones, such as H3K18, H4K5, and H4K18 in macrophages [24]. H3K14la has been reported to be associated with microtubule movement and cell invasion in Toxoplasma [44]. Here, our data demonstrate a significant increase in H3K14la level in PCNs since 8 h after hemin stimulation, which was confirmed in vivo where the levels of neuronal H3K14la were increased in the perihematomal region in ICH mice. Importantly, we observed that the H3K14la level correlated with the process of neuronal ferroptosis upon hemin treatment, and that inhibition of H3K14la suppressed hemin-induced neuronal ferroptosis and improved the outcomes after ICH. This is the first study to observe histone lactylation driving neuronal ferroptosis and indicates the potential of H3K14la as a therapeutic target in ICH.

Previous studies have found that lactylation plays a role in transcriptional activation [24], but in this study, H3k14la downregulates several related genes, including *PMCA2*, which encodes PMCA2 pumping intracellular Ca^2+^ out of cells. A previous study showed that at the early stage of ICH, activation of Ca^2+^ channel receptors leads to elevation of Ca^2+^ in neurons, which participated in ICH progression [45]. A recent study showed that Ca^2+^ overload accelerates the secondary cerebral damage triggered by ICH [46]. Moreover, increased cytosolic calcium is a hallmark of ferroptosis, which can be inhibited by a ferroptosis inhibitor Fer-1, before complete cell lysis [6, 42]. This study suggested that H3K14la promoted ferroptosis by downregulating *PMCA2* and resulting in subsequent intracellular Ca^2+^ accumulation. We expounded on the mechanisms and role of Ca^2+^ overload in the disease progression of ICH via ferroptosis.

In summary, our study showed for the first time that histone lactylation levels were elevated in neuronal ferroptosis post-ICH and that the inhibition of histone lactylation was effective in rescuing neuronal ferroptosis, providing a novel potential therapeutic candidate for ICH treatment. Additionally, we also demonstrated that H3K14la regulated neuronal ferroptosis by decreasing *PMCA2* in vitro and in vivo.

## Materials and methods

### Animals

All animal studies were carried out under the Guide for the Care and Use of Laboratory Animals, and approved by the Animal Care and Use Committee of Capital Medical University. All neonatal mouse pups (P0-2) and C57BL/6 male mice (6–8 weeks old) used in this study were from Vital River (China). The animals were kept under a 12-h light/dark cycle with accessible water and food. Briefly, the mice were anesthetized using 1–2% isoflurane inhalation and fixed in a stereotaxic apparatus (E04545, RWD). Samples were excluded from the mice that were died. Mice per group were randomized with the website www.randomization.com, and all the treatments, as well as the data collection were blinded by different investigators.

### Primary neuronal cultures

C57BL/6 mouse pups (postnatal 0–1 d) were disinfected in 75% ethanol. The brains were removed and decapitated, and the heads were collected in Hank’s solution (H1045-500, Solarbio) on ice. The dissected cortices were cut into 1 mm pieces in 0.5 mL DNase I (200 μg/mL, D8071, Solarbio), and digested by papain working solution (Daspase II 1.2 U/mL + papain 1 mg/mL; Daspase II 04942078001, Roche; papain, P8150, Solarbio). After centrifuging at 1000 rpm for 10 min and discarding the supernatant, the cells were re-suspended with DMEM (11039021, Gibco), including 10% fetal bovine serum (10099141C, Thermo). In the following of filtered Supernatant cells through 70 µm mesh filters (352350, Falcon), cells were counted and seeded in the plate treated with poly-D-lysine (P0899, Sigma-Aldrich). Cytarabine (PHR1787, Sigma-Aldrich) was to remove glial cells 24 h later and half of the medium was changed every 3 days. Primary Cortical Neurons (PCNs) were cultured in Neurobasal medium (21103049, Gibco) containing 2% B27 (17504044, Invitrogen), 1% glutamine (G8540, Sigma), and 1% penicillin-streptomycin (30-002-CI, Corning) at 37 °C, 5% CO_2_, 95% saturated humidity in an incubator for 7–10 d. We randomized the cell culture group using the website www.randomization.com. All the experimental conditions and quantifications for the cell cultures were blinded by masking labels or using different investigators.

For viral transduction, 1.5 × 10^5^ cells were transduced with 0.5 µL of AAV-hSyn-EGFP- NC (titer: 2.81 × 10^12^ vg/ml) or AAV-hSyn-EGFP-p300.DN (titer: 2.98 × 10^12^ vg/ml) at 4 h after seeding.

### Drug administration

Unless specifically indicated, PCNs were exposed to 50 µM hemin (16009-13-5, Frontier Scientific), 2 µM Fer-1 (S7243, Selleck), 50 µM DFO (D9533, Sigma-Aldrich), DMSO (D8418, Sigma), 50 µM GSK 2837808 A (5189/10, Tocris), 10 mM Sodium oxamate (S6871, Selleck), A485 (0.05, 0.2, 1, 5 µM. HY-107455, MCE), Apicidin (0.2, 1, 5, 10 µM. HY-N6735, MCE) and Caloxin 2a1 (0.2, 0.4, 0.8, 1 mM. HY-P3278A, MCE) for 24 h.

To induce the ICH mouse model, each mouse was injected with 0.5 µL of collagenase VII-S (0.1 U/μL, Sigma-Aldrich) in the left striatum at the following coordinates: x = 2.0 mm; y = 0.8 mm; z = −3.15 mm [47]. Sham mice received a needle insert. At 30 min after collagenase injection, each mouse was injected with 1 μl of 500 μM GSK or 10 mM Caloxin 2A1.

### PI and Hoechst staining

PCNs (1.5 × 10^5^) were seeded in 48-well plates for 7 d as described previously [19]. After treatment with drugs, PCNs were incubated with 20 µg/mL propidium iodide (PI) dye (P4170, Sigma-Aldrich) and Hoechst 33342 (1:1000. C0031, Solarbio) for 30 min in the incubator. The fluorescence microscope (Nikon ECLIPSE Ti) was used to take pictures, and the number of PI^+^ cells divided by the total number of cells was used as the percentage of cell death.

### Lipid peroxidation measurement

Lipid ROS were detected by BODIPY™ 581/591 C11 reagent with a fluorescence microscope (D3861, Invitrogen). PCNs (1.5 × 10^5^) were seeded in 48-well plates for 7 d as described previously and treated with drugs for 24 h. According to the manufacturer’s instructions, PCNs were incubated with 10 μM reagent dye for 30 min in the incubator. Fluorescence microscopy was used to detect the fluorescence intensity, and results were analyzed by image J. The ratio of 590 nm to 510 nm was used to represent the intracellular ROS level.

### Western blot

For Western blots of the striatum, mice were sacrificed at 24 h after ICH. For the western blot of PCNs, PCNs (2 × 10^6^) were seeded in 6-well plates. The dissected striatum or cells were homogenized RIPA lysis solution (C1053, Applygen) including protease cocktail inhibitors (4693132001, Roche) for 30 min, protein concentration was detected by BCA protein assay reagent (23228, Thermo Fisher Scientific). The 20 µg/µL protein was isolated by 12% SDS-PAGE (B1005, Applygen) electrophoresis before being transferred to the PVDF membrane (1620177, Millipore). The target protein was bound with primary antibodies and secondary antibodies. The blots were detected by a chemiluminescence imager (Fusion SoloS. EDGE, VILBER) using the Immobilon Wester chemilum HRP substrates (WBKLS0500, Millipore). Western blot was incubated with the following antibodies: anti-Histone H3 (mono methyl K9) antibody (1:10,000, ab9045, Abcam), anti-Histone H3 (di methyl K9) antibody (1:10,000, ab1220, Abcam), anti-Histone H3 (tri methyl K9) antibody (1:10,000, ab8898, Abcam), anti-Histone H3 (mono methyl K27) antibody (1:10,000, ab194688, Abcam), anti-Histone H3 (di methyl K27) antibody (1:10,000, ab24684, Abcam), anti-Histone H3 (tri methyl K27) antibody (1:10,000, ab192985, Abcam), anti-acetyl Lysine antibody (1:1000, ab80178, Abcam), anti-Histone H3 (mono methyl K4) antibody (1:1000, GTX54100, GeneTex), anti-Histone H3 (di methyl K4) antibody (1:1000, ab32356, Abcam), anti-Histone H3 (tri methyl K4) antibody (1:1000, ab8580, Abcam), Anti-L-Lactyl Lysine rabbit mAb antibody (1:1000, PTM-1401RM, PTM BIO), anti-L-Lactyl-Histone H3 (Lys14) rabbit mAb (1:1000, PTM-1414RM, PTM BIO), anti-L-Lactyl-Histone H4 (Lys8) rabbit mAb (1:1000, PTM-1415RM, PTM BIO), anti-L-Lactyl-Histone H4 (Lys5) rabbit mAb (1:1000, PTM-1407RM, PTM BIO), anti-H3 antibody (1:2000, ab10799, Abcam), anti-H4 antibody (1:2000, PTM-1009RM, PTM BIO), anti-spectrin (1:500; BML-FG6090; Enzo), anti-rabbit IgG, HRP-linked antibody (1:1000, 7071S, CST, USA), anti-mouse IgG, HRP-linked antibody (1:1000, 7076S, CST), anti-4-HNE (1:1000, MAB3249, R&D system).

### Dot blot

DNA concentration was detected after extraction and diluted to 500 ng/100 μL with autoclaved water. Each well was sampled with 100 μL DNA solution. After membrane washing, ultraviolet crosslinking, and sealing, antibody incubation and image collection were carried out. Dot blot was incubated with the following antibodies: anti-5-methylcytosine (5-mC) antibody (2 µg/mL, ab214727, Abcam), anti-5-hydroxymethylcytosine (5-hmC) antibody (1 µg/mL, ab214728, Abcam).

### siRNA transfection

PCNs were cultured for 3–4 days in the incubator and then transfected with siRNA. Briefly, LipofectamineTM RNAiMAX (1:500, 13778150, Invitrogen) and 50 µM siRNA were respectively added to the Opti-MEMTM reduced serum culture (31985070, Gibco) for incubation at room temperature for 5 min, and then mixed for 5 min according to instruction. The medium in the culture plate was discarded and Opti-MEMTM with siRNA was added for 4–6 h in the incubator before changing the medium. The sequences of siRNAs are as follows: siRNA LDHA-1 (F) CGUCUCCCUGAAGUCUCUUAATT, (R) UUAAGAGACUUCAGGGAGACGTT; siRNA LDHA-2 (F) GCUUGUGCCAUCAGUAUCUUATT, (R) UAAGAUACUGAUGGCACAAGCTT; siRNA LDHA-3 (F) CCAGCAAAGACUACUGUGUAATT, (R) UUACACAGUAGUCUUUGCUGGTT; siRNA LDHB-1 (F) CCCAGUGGAUAUUCUGACUUATT, (R) UAAGUCAGAAUAUCCACUGGGTT; siRNA LDHB-2 (F) CGUCAUCAAUCAGAAGCUGAATT, (R) UUCAGCUUCUGAUUGAUGACGTT; siRNA LDHB-3 (F) CCGAACAACAAGAUCACUGUATT, (R) UACAGUGAUCUUGUUGUUCGGTT; siRNA FBXW7-1 (F) CGCAUAGUUAGUGGUUCUGAUTT, (R) AUCAGAACCACUAACUAUGCGTT; siRNA FBXW7-2 (F) CGGAGGAUUACAUCUGUCCAATT, (R) UUGGACAGAUGUAAUCCUCCGTT; siRNA FBXW7-3 (F) CCAGUGUUUACACGUCCUGAUTT, (R) AUCAGGACGUGUAAACACUGGTT.

### RT-PCR

Total mRNA was extracted from PCNs (1 × 10^6^ per well in 24-well plates) or the striatum (24 h after ICH) by TRIzol reagent (15596026, Life Technologies, USA). cDNA was reversed with a Reverse Transcriptase kit (R223-01, Vazyme) according to the manufacturer’s instructions. The real-time quantitative PCR kit was PowerUpTMSYBRTM Green Master Mix (A25742, Applied Biosystem), detected by ABI 7500 Fast Real-Time Fluorescent Quantitative PCR system (Applied Biosystem 7500). The primers used are as follows: *Ptgs2*: (F) TGAGCAACTATTCCAAACCAGC, (R) GCACGTAGTCTTCGATCACTATC; *FBXW7*: (F) TGCAAAGTCTCAGATTATACC, (R) TTTCTCTCTCCAGAGAAGGTTATC; *ZEB1*: (F) TGGCAAGACAACGTGAAAGA, (R) AACTGGGAAAATGCATCTGG; *TFR2*: (F) TTGGGGTCTACTTCGGAGAGT, (R) GACAGGAGCCTAAGTGCTCAG; *LDHA*: (F) TGTCTCCAGCAAAGACTACTGT, (R) GACTGTACTTGACAATGTTGGGA; *LDHB*: (F) CATTGCGTCCGTTGCAGATG, (R) GGAGGAACAAGCTCCCGTG; *PMCA2*: (F) GGTGACATGACCAACAGCGA, (R) CCCCATACGTCTCCTTGATCT.

### ChIP and ChIP-seq

The N2A cell line is well-used to mimic primary neurons [48]. The N2A cells were cultured in DMEM high glucose medium (C11995500BT, Gibco) including 10% fetal bovine serum (FBS, 10099141, Gibco) and 1% penicillin–streptomycin mixture (KGY0023, Keygen) in a humidified incubator at 37 °C and 5% CO_2_. Cells were passaged every 3 days. N2A cells (1 × 10^7^) were seeded in 150 mm cell culture dishes. After drug administration, N2A cells were cleaned with PBS and fixed with formaldehyde at 37 °C for 10 min (F8775, Sigma-Aldrich). In the following scraping cells, the DNA was treated into small fragments with an ultrasonic lysis instrument (Time: 00:00:30, Pulse: Amp1 45%). The DNA was incubated with H3K14la (PTM-1414RM, PTM BIO) or IgG (C1755, Applygen) antibody overnight, and immunoprecipitation was performed with protein A/G beads (sc-2003, Santa Cruz). After multiple elution processes, the DNA was purified with a PCR Purification Kit (28106, QIAquick). Real-time PCR (RT-PCR), DNA electrophoresis experiments, and ChIP-seq were conducted subsequently. The DNA sequencing process was conducted by Beijing iGene Code Biotec (Beijing, China) on the MGISEQ T7 platform. The primer sequence of the FBXW7 fragment used in the ChIP experiment is as follows: *FBXW7*-primer1 (F) GGGAATGCTGCTGTAGTT3, (R) GGTAAGCTCATAATATGGCTC; *FBXW7*-primer2: (F) ATAACTTGGCTGTGAGTGAGA, (R) CAGATTTGTGGCTTCCTTT; *PMCA2*-primer1 (F) CTATTCACCCTGGCTACTGG, (R) CGGCTAAAGGCTGTTGG; *PMCA2*-primer2: (F) CAGCCAGGCACCACAGA, (R) CAGGTATTGGGACAGGATTT.

### RNA-sequencing

PCNs were treated with Veh (DMSO) and 50 µM hemin for 12 h or 24 h and then collected for RNA-seq analysis (Beijing igeneCode Biotech Co. Ltd, Beijing, China). RNA-Seq analysis was performed with the Illumina HiSeq platform to identify the differential expression genes.

### Viruses injection

Stereotaxic surgery was performed after mice were anesthetized with an isoflurane vaporizer. To knockdown *p300 in* striatal neurons, each mouse received 2.4 µL of AAV-hSyn-EGFP-NC (titer: 2.81 × 10^12^ vg/ml) or AAV-hSyn-EGFP-shP300 (titer: 2.98 × 10^12^ vg/ml) at the following 4 different stereotaxic coordinates with respect to bregma: x = 2.2 mm, y = 0.6 mm, z = −3.05/−3.25; x = 1.8 mm y = 1 mm and z = −3.05/−3.25. The injection speed was 0.1 µL/min and the needle was showly removed 10 min after injection.

### Metabolomics analysis

The metabolomics analysis was carried out by Biotree Technologies, China. After mouse brains were extracted in the solution (methanol: water = 3:1, with the isotopically-labeled internal standard mixture), the mixture was centrifuged at 13,800 g for 15 min at 4 °C. The collected supernatant was used for LC-MS/MS analysis. LC-MS/MS analysis was carried out using a UHPLC system (Thermo Fisher Scientific vanquish, USA) with a UPLC HSS T3 column coupled with Orbitrap exploris 120 mass spectrometer (Orbitrap MS, Thermo Fisher Scientific). MS/MS spectra acquisition was performed using Orbitrap Exploris 120 mass spectrometer. An in-house program was used to analyze the original data being converted to the mzXML format. For metabolite annotation, the internal MS2 database (biotreedb) was applied and the cutoff for annotation was 0.3.

### Immunofluorescence and FJC staining

At 24 h after ICH, mice were anesthetized and perfused. For immunofluorescence analysis, the frozen brain sections were prepared and then incubated in an antigen-repair solution. After permeabilization with 1% Triton X-100 for 15 min, sections were blocked with 5% goat serum. Afterwards, sections were incubated with primary antibodies against histone lysine lactylation (Kla) (1:100, PTM-1401RM, PTM BIO), H4K8 (1:100, PTM-1415RM, PTM BIO), L-Lactyl-Histone H3 (Lys14) rabbit mAb (1:100, PTM-1414RM, PTM BIO) and NeuN (1:500, ABN91, Merk) at 4 ^◦^C overnight. After further rinsing, sections were incubated with secondary antibodies for 2 h at RT and then stained with a mounting medium containing DAPI (ZG1202, Vectarshield). Three areas in the perihematomal region per section were taken with a confocal microscope (TCS SP8 STED, Leica), and the immunofluorescence intensity was measured by Image J software. For FJC staining, the frozen brain sections were incubated in a solution containing 1% sodium hydroxide (NaOH) in 80% ethanol for 2 min, followed by an incubation in 70% alcohol for 2 min. After rinsing, the sections were treated with 0.06% potassium permanganate for 10 min. Following another wash, the sections were stained with 0.0004% FJC (AG325, Millipore). 3 areas per section around the lesion site were imaged with a fluorescence microscope (ECLIPSE Ti–U, NIKON) at an excitation wavelength of 450–490 nm. The number of FJC^+^ cells was counted by Image J software.

### Assessment of neurologic deficit score

As described previously [12], neurologic deficit scores were determined by six neurologic tests including body symmetry, climbing, gait, front limb symmetry, circling behavior, and compulsory circling at 6 h, 12 h, and 24 h after ICH in mice. Each test was scaled from 0 to 4, and 0 represented the slightest impairment of mice.

### Wire hanging test

Wire hanging test was performed with a stainless-steel rod (diameter: 2 mm; length: 50 cm). The mice were placed in the middle of the stainless-steel rod and observed for 30 s each time for four trials. The test was scored according to the following criteria: 0 for falling off; 1 for hanging onto the rod with two forepaws; 2 for hanging onto the rod with two forepaws and attempting to climb onto the rod with one or two hind paws; 3 for hanging onto the rod with two forepaws as well as one or both hind paws; 4 for hanging onto the rod with four paws; 5 for climbing to one of the supports.

### Hindlimb placing test

The mouse was placed on the edge of the table, and then the right hind limb was pulled down. Behaviors were scored as follows: 0 point (immediate retraction); 1 point (delayed retraction); 2 points (unable to retract). The test scores were quantified as the sum of 10 trials.

### Immunocytochemistry

Following fixation with 4% paraformaldehyde (PFA) at RT for 10 min, PCNs were permeabilized in 0.3% TBST at RT for 20 min. Subsequently, the cells were blocked with 10% BSA at RT for 1 h, and then incubated with primary antibodies against NeuN (1:500, ABN91, Merk) and Tau (1:200, MAB3420, Millipore) at 4 ^◦^C overnight. After further permeabilization, cells were incubated at RT for 1 h with secondary antibodies. Finally, nuclei were stained with DAPI (ZG1202, Vectarshield). Photographs were imaged using a confocal microscope (Leica DM6000CS Microsystems).

### Statistical analysis

All data are shown as mean ± SD unless otherwise stated and all statistical analyses were performed with GraphPad Prism software (version 8.0). Statistical analysis was performed using unpaired two-tailed Student’s *t* test for two groups, while one-way ANOVA or two-way ANOVA followed by Tukey’s multiple comparisons tests or Dunnett’s test was performed for comparisons of multiple groups. *p* < 0.05 was considered statistically significant.

## Supplementary information

Supplementary figures

original western blots

## Data Availability

All the data in this study are available from the corresponding author on reasonable request.
