# Supplementary material for: H3K14 lactylation exacerbates neuronal ferroptosis by inhibiting calcium efflux following intracerebral hemorrhagic stroke
Source: Cell Death Dis. 2025 Jul 23;16(1):553. doi: 10.1038/s41419-025-07874-9 (PMC12287320; doi:10.1038/s41419-025-07874-9)
Supplement: Supplementary file 1 — Supplementary figures [file 41419_2025_7874_MOESM1_ESM.docx]

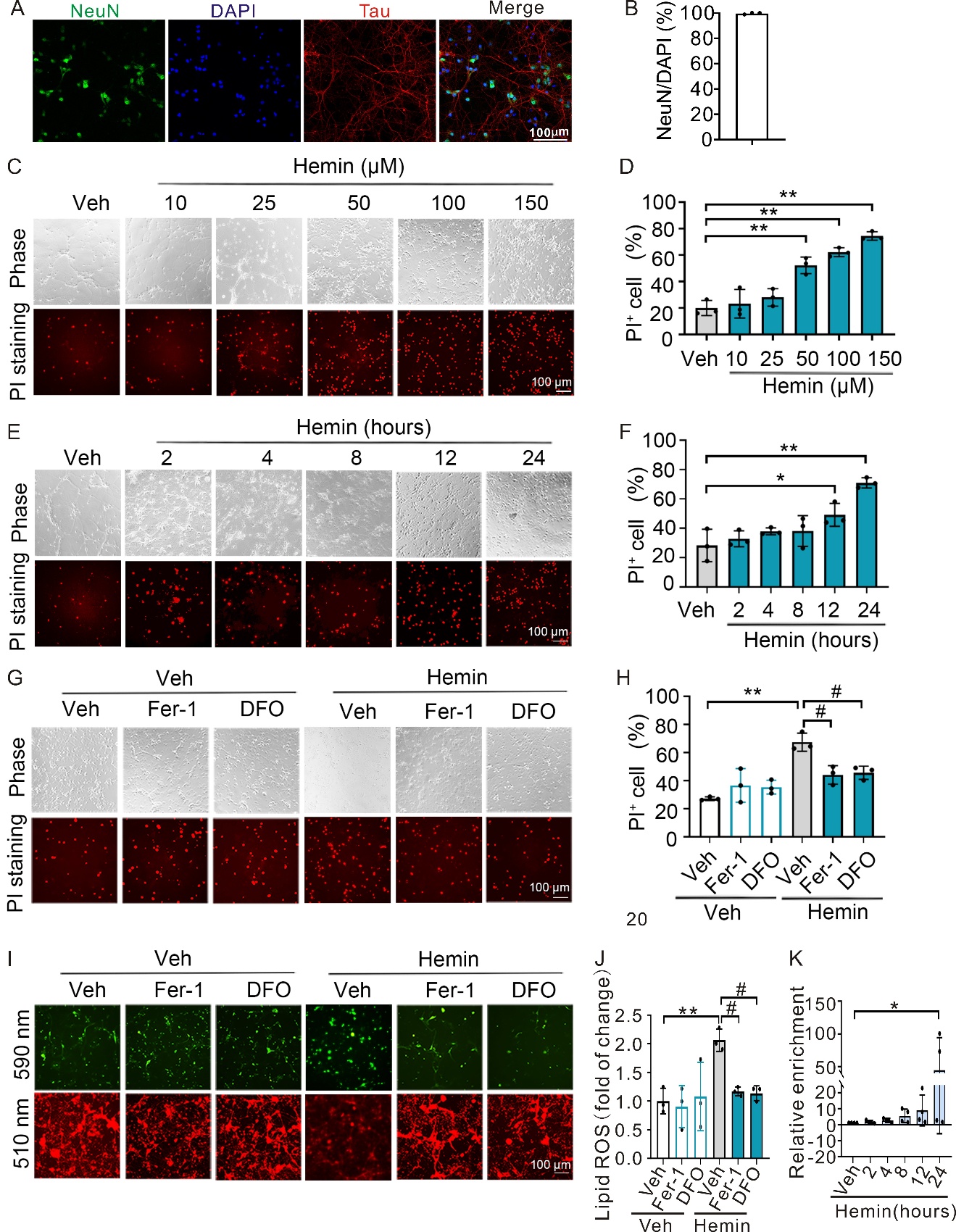


**Fig. S1**. **Hemin induces ferroptosis in primary cortical neurons *in vitro*. (A, B)** The staining of Tau and NeuN was performed to confirm the purity of primary cortical neurons (PCNs). n = 3 cultures. **(C, D)** PCNs were treated with different concentrations of hemin or Veh (0.75% DMSO). PI staining was performed to detect cell death. n = 3 cultures. **(E, F)** PCNs were treated with 50 µM hemin at the indicated time points. PI staining was performed to detect cell death. n = 3 cultures. **(G, H)** Ferroptosis inhibitors Fer-1 (2 µM) or 50 µM DFO were treated to PNCs with hemin for 24 h, and cell death was detected by PI staining. n = 3 cultures. **(I, J)** PCNs were treated with hemin and Fer-1 or DFO, and lipid ROS were detected by the BODIPY 581/591 C11 reagent. n = 3 cultures. **(K)** RT-PCR detected *Ptgs2* mRNA levels in PCNs treated with hemin at different time points. n = 4 cultures. GAPDH serves as the internal control. Results are shown as scatter plots (Mean±SD). One-way ANOVA followed by Dunnett’s (D, F, K) or Tukey's (H, J) multiple comparisons tests was used. **p* < 0.05, ***p* < 0.01 *vs* Veh; #*p* < 0.05 *vs* Hemin.


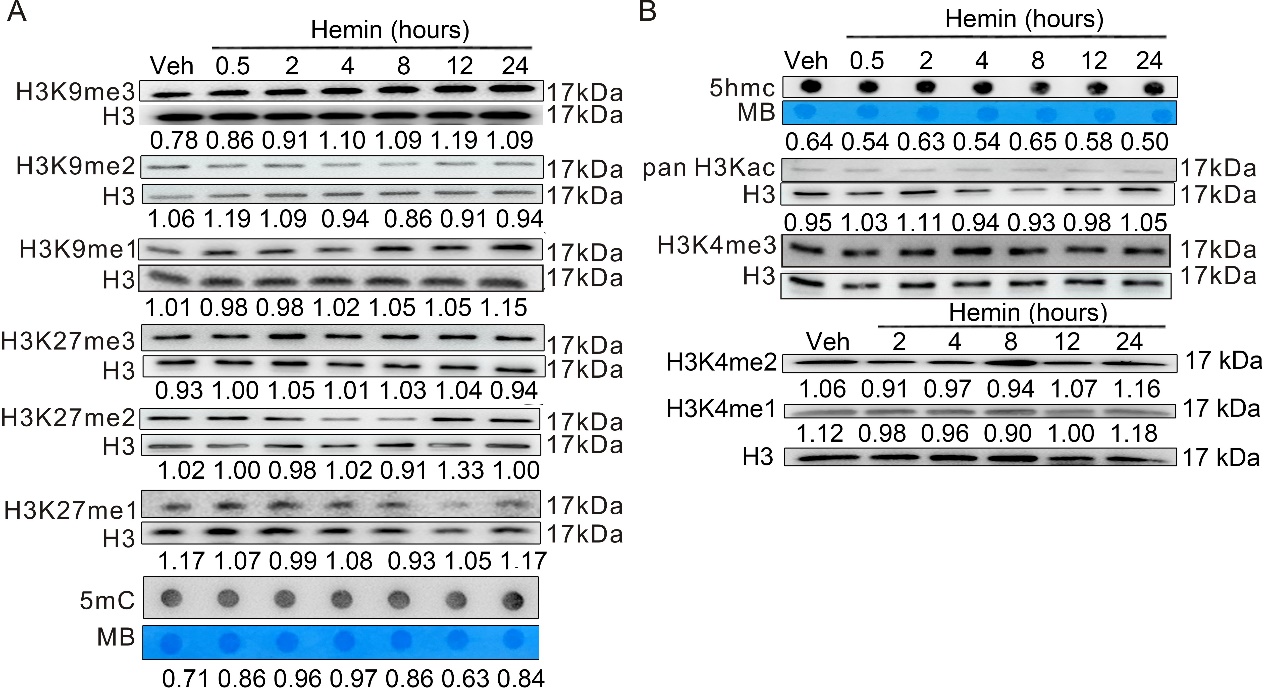


**Fig. S2. Characterization of the transcriptional activation and repression markers in primary cortical neurons after hemin treatment *in vitro*.** (A-B) PCNs were treated with 50 µM hemin, and the expression of the indicated modifications was detected by Western blotting or dot blotting. H3, H4, or methylene blue as the internal control. 5mc: 5-methylcytosine; 5hmc; 5-hydroxymethylcytosine.


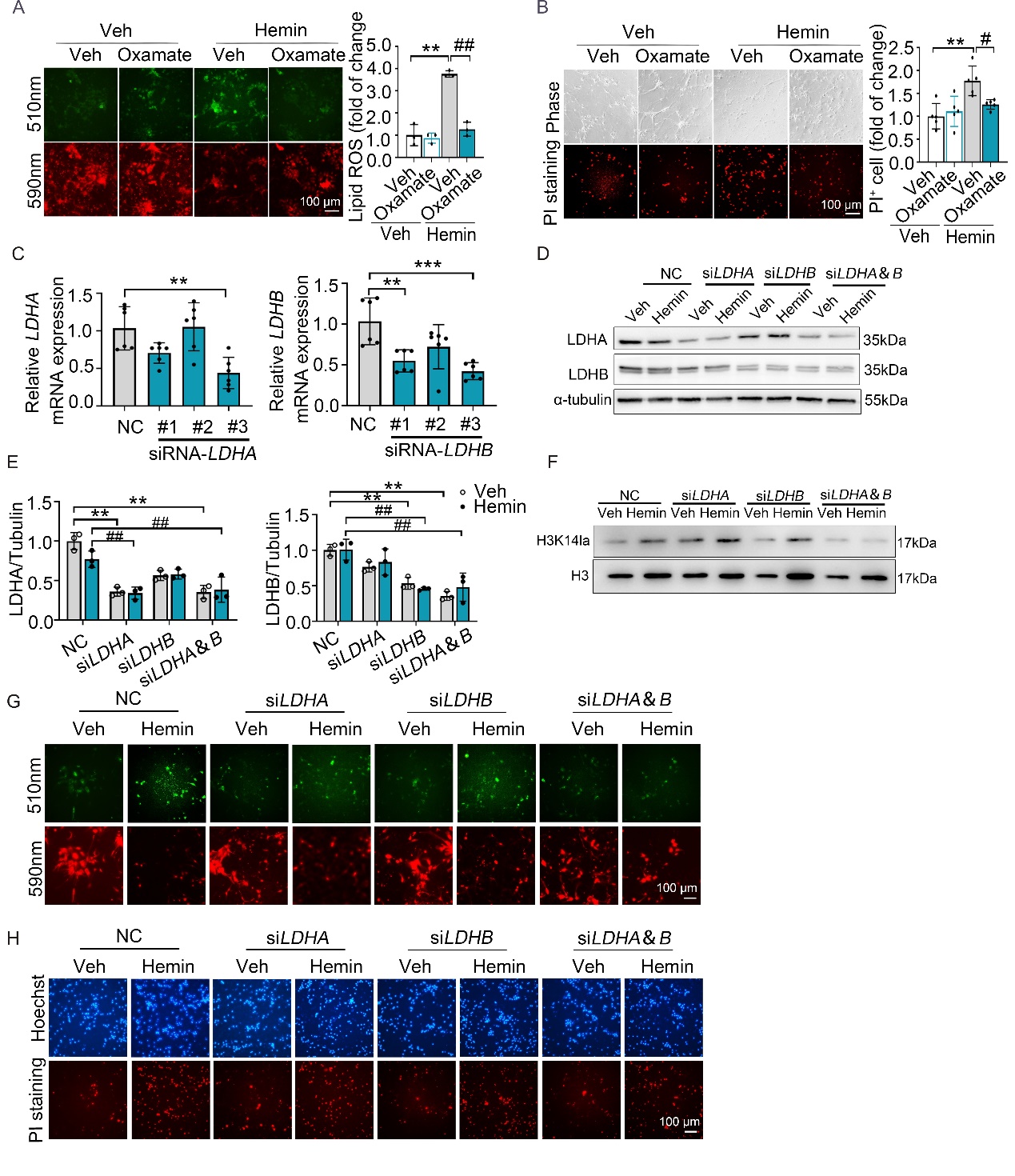


**Fig. S3. Inhibition of LDH activity attenuated hemin-induced neuronal ferroptosis *in vitro*.** **(A)**The BODIPY 581/ 591 C11 reagent was used to detect the changes of intracellular lipid ROS later at 24 h after treatment with 50 µM hemin and 10 mM oxamate. n = 3 cultures. **(B)** The changes in cell death after 50 µM hemin and 10 mM oxamate combined treatment later 24 h were detected by PI staining. n = 5 cultures. **(C)** After transfection of three siRNA sequences of LDHA and LDHB in PCNs, mRNA levels of LDHA or LDHB were detected by PCR. GAPDH serves as the internal control. n = 6 cultures. **(D, E)** The protein expression of LDHA and LDHB was detected by Western blotting after transfection siRNA of LDHA and LDHB. α-tubulin serves as an internal control. n = 3 cultures. **(F)** The representative images show Western blotting of H3K14la in PCNs after transfection siRNA of LDHA and LDHB at 12 h after 50 µM hemin treatment. **(G-H)** The representative images show the lipid ROS (G) or cell viability (H) in PCNs after transfection siRNA of LDHA and LDHB at 12 h after 50 µM hemin treatment. Results are shown as scatter plots (Mean±SD). One-way ANOVA followed by Tukey's multiple comparisons tests was used. **p < 0.01, ***p < 0.001 *vs* Veh/NC; ## p <0.01 *vs* Hemin.


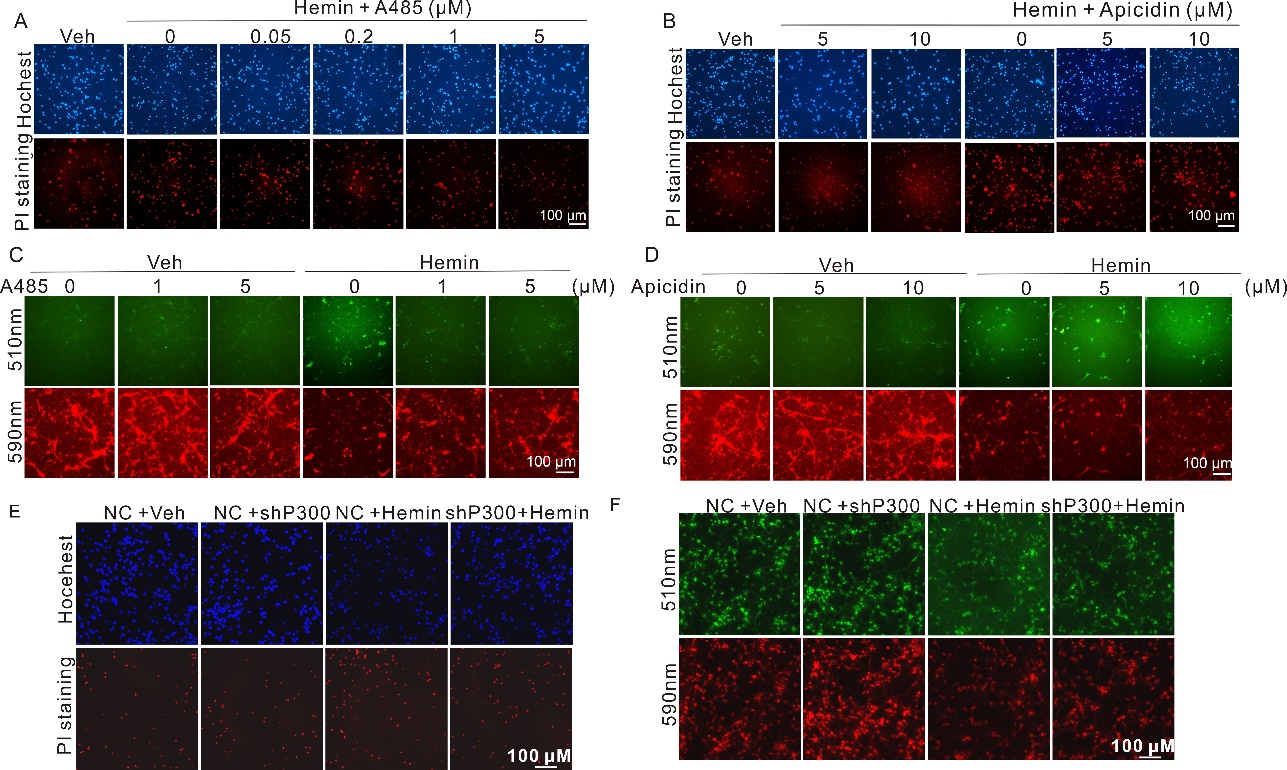


Fig. S4. Role of P300/CBP and class I HDAC on neuronal ferroptosis induced by hemin *in vitro.* (A, B) The representative images show the changes in cell death in PCNs at 24 h after co-treatment of hemin with A485 or Apicidin. (C, D) The representative images show the changes of intracellular lipid ROS in PCNs at 24 h after co-treatment of hemin with A485 or Apicidin. (E) The representative images show the cell death of PCNs transduced with the AAV-hSyn-EGFP-NC (NC) or AAV-hSyn-EGFP-shP300 (shP300) at 24 h after hemin treatment. (F) The representative images show the changes of intracellular lipid ROS in PCNs transduced with AAV virus NC or shP300 at 24 h after hemin treatment.


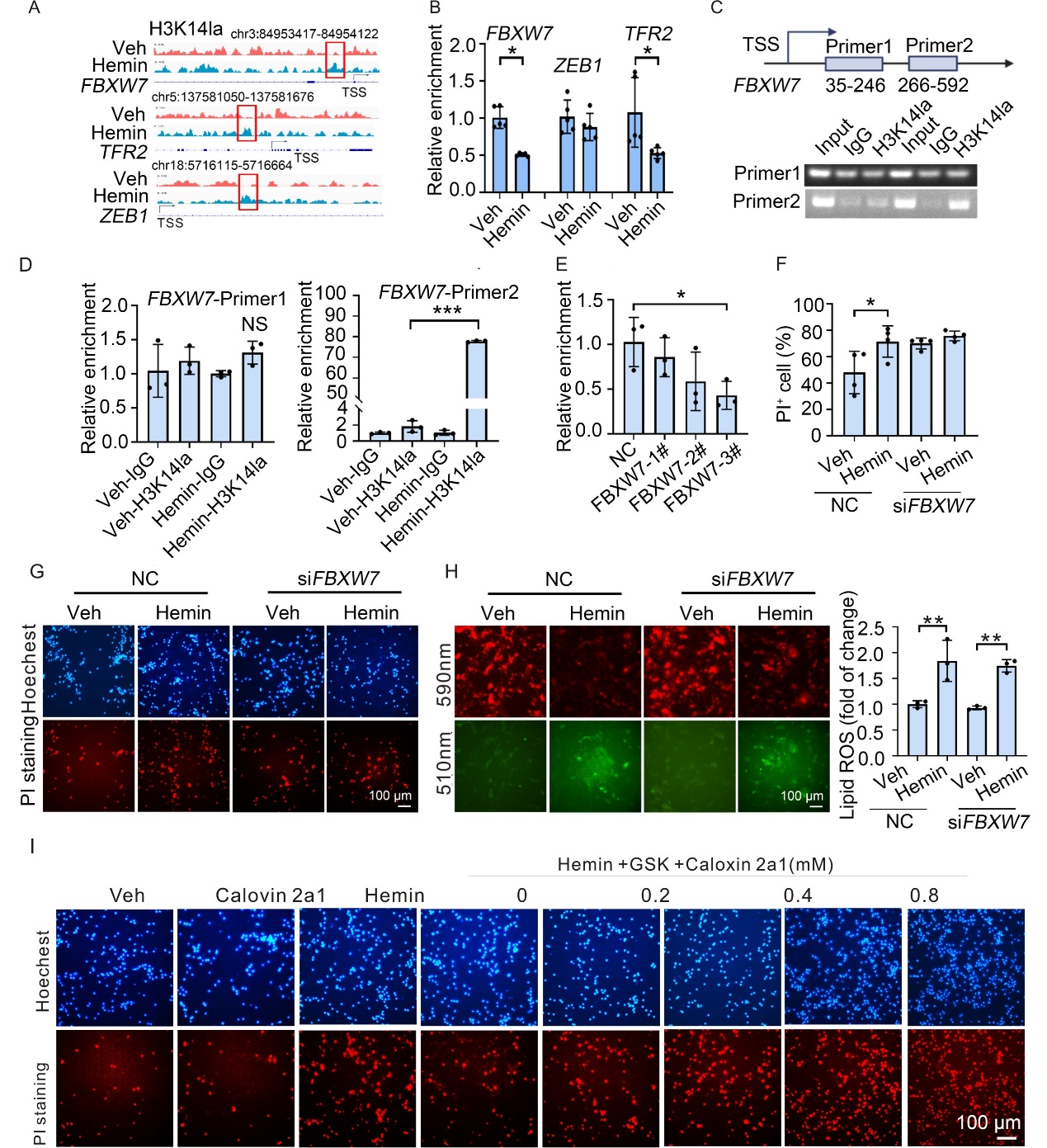


**Fig. S5. Identification of the downstream targets of H3K14la in neuronal ferroptosis induced by hemin *in vitro*.** **(A)** The peaks of target genes were analyzed by IGV. The red and blue [respectively](javascript:;) indicate the peak regions of H3K14la on target-gene in Veh and hemin groups. **(B)** mRNA levels of genes (*FBXW7/TFR2/ZEB1*) were detected by RT-PCR after 50 µM hemin treatment of PCNs at 12 h. n = 5 cultures. GAPDH serves as the internal control. **(C-E)** The [enrichment](javascript:;) of H3K14la in *FBXW7* was detected by ChIP-PCR in Veh and hemin treatment groups in N2A cells. n = 3 cultures. **(F, G)** After transfecting PCNs with *FBXW7* siRNA, the cell viability of Veh and hemin groups was detected by PI and Hoechst staining. n = 4 cultures. **(H)** After transfecting PCNs with *FBXW7* siRNA, lipid ROS of Veh and hemin groups was detected. Results are shown as scatter plots (Mean ± SD). n = 3 cultures. **(I)** The representative images show the changes in cell death of PCNs after 50 µM hemin and Caloxin 2a1 (Cal) combined treatment later 24 h by PI and Hoechst staining. n = 3-5 independent experiments. Unpaired two-tailed Student’s t-test (B), one-way ANOVA followed by Tukey's (D, F, H) or Dunnett’s (E) multiple comparisons tests were used. *p < 0.05, **p < 0.01, ***p < 0.001 *vs* Veh/NC; NS, not significant.
