# Supplementary figures and images for: H3K14 lactylation exacerbates neuronal ferroptosis by inhibiting calcium efflux following intracerebral hemorrhagic stroke
Source: Cell Death Dis. 2025 Jul 23;16(1):553. doi: 10.1038/s41419-025-07874-9 (PMC12287320; doi:10.1038/s41419-025-07874-9)

Fig.1E

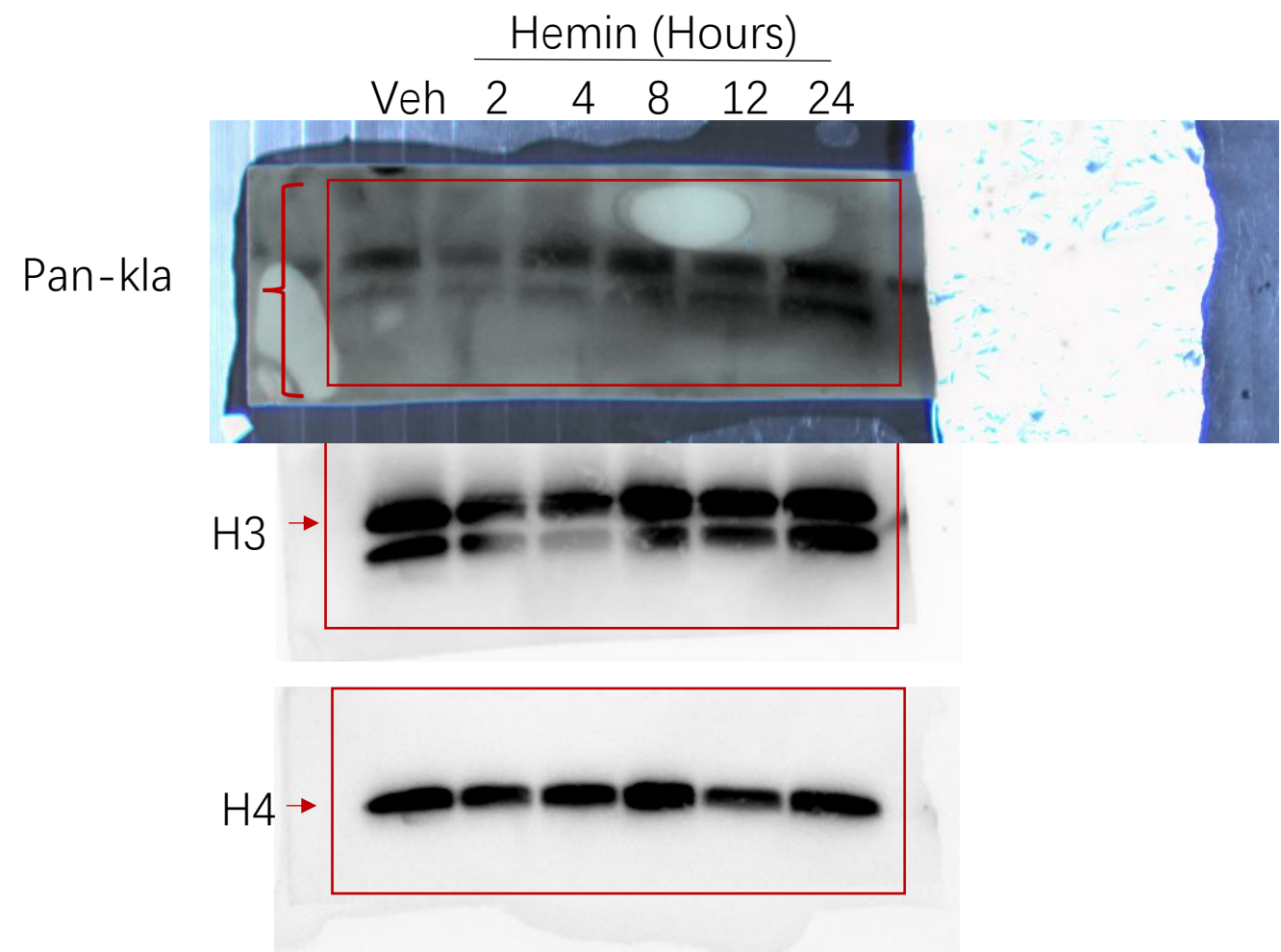

Fig.1F

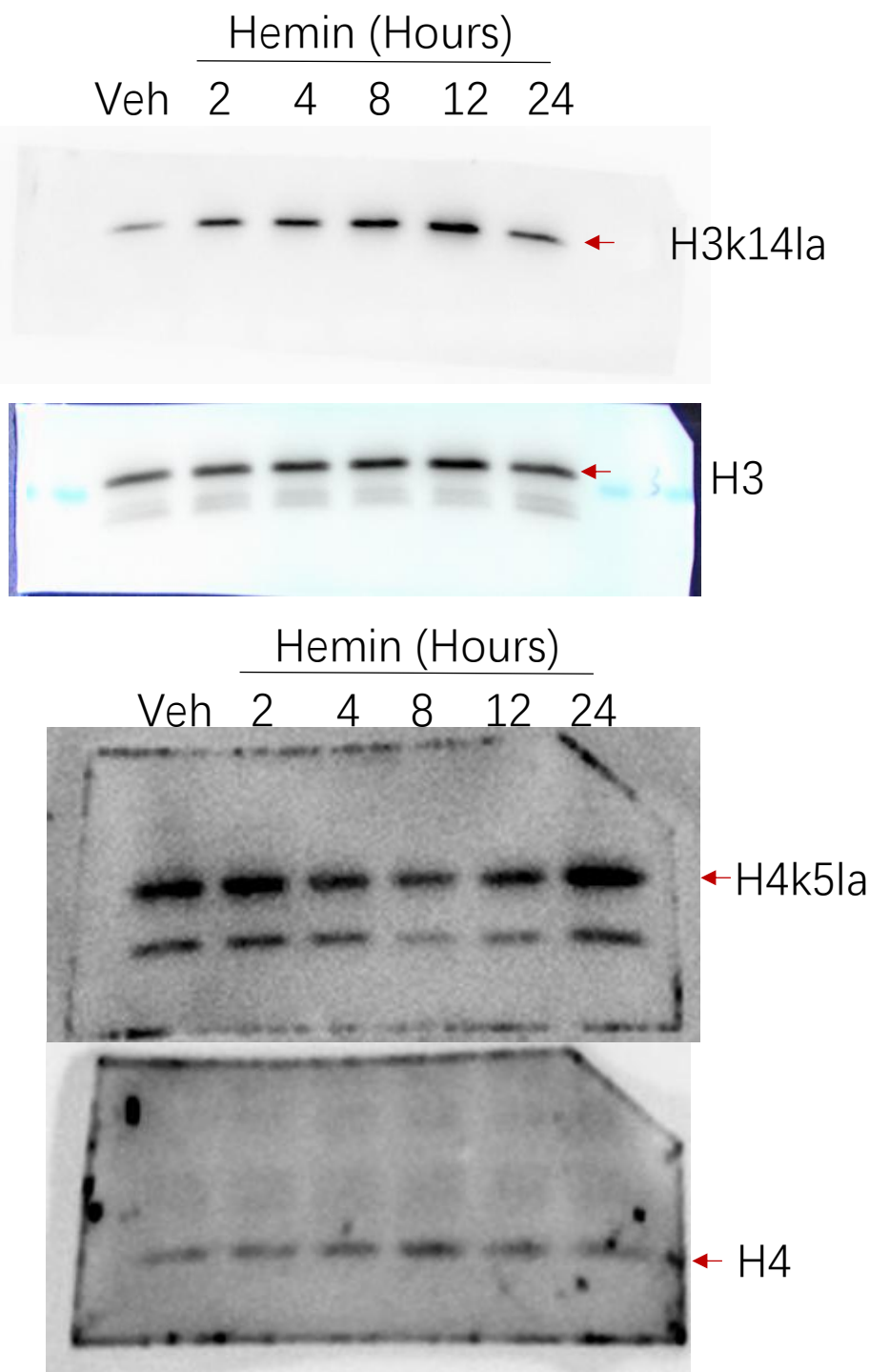

Fig.1F

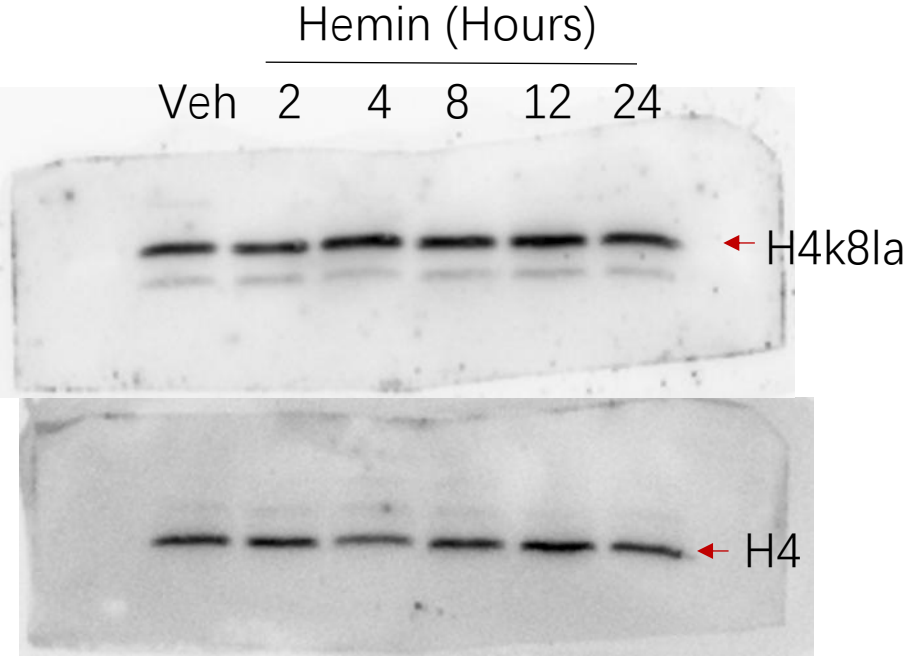

Fig.2A

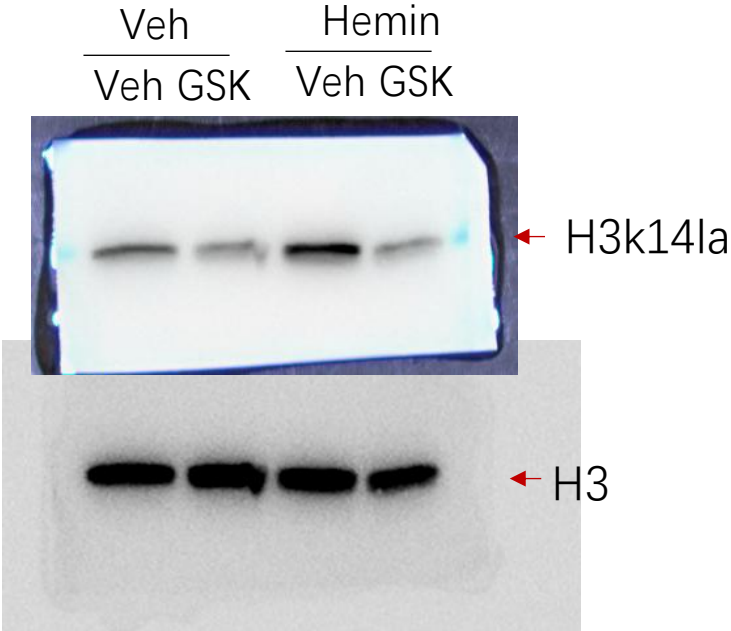

Fig.2A

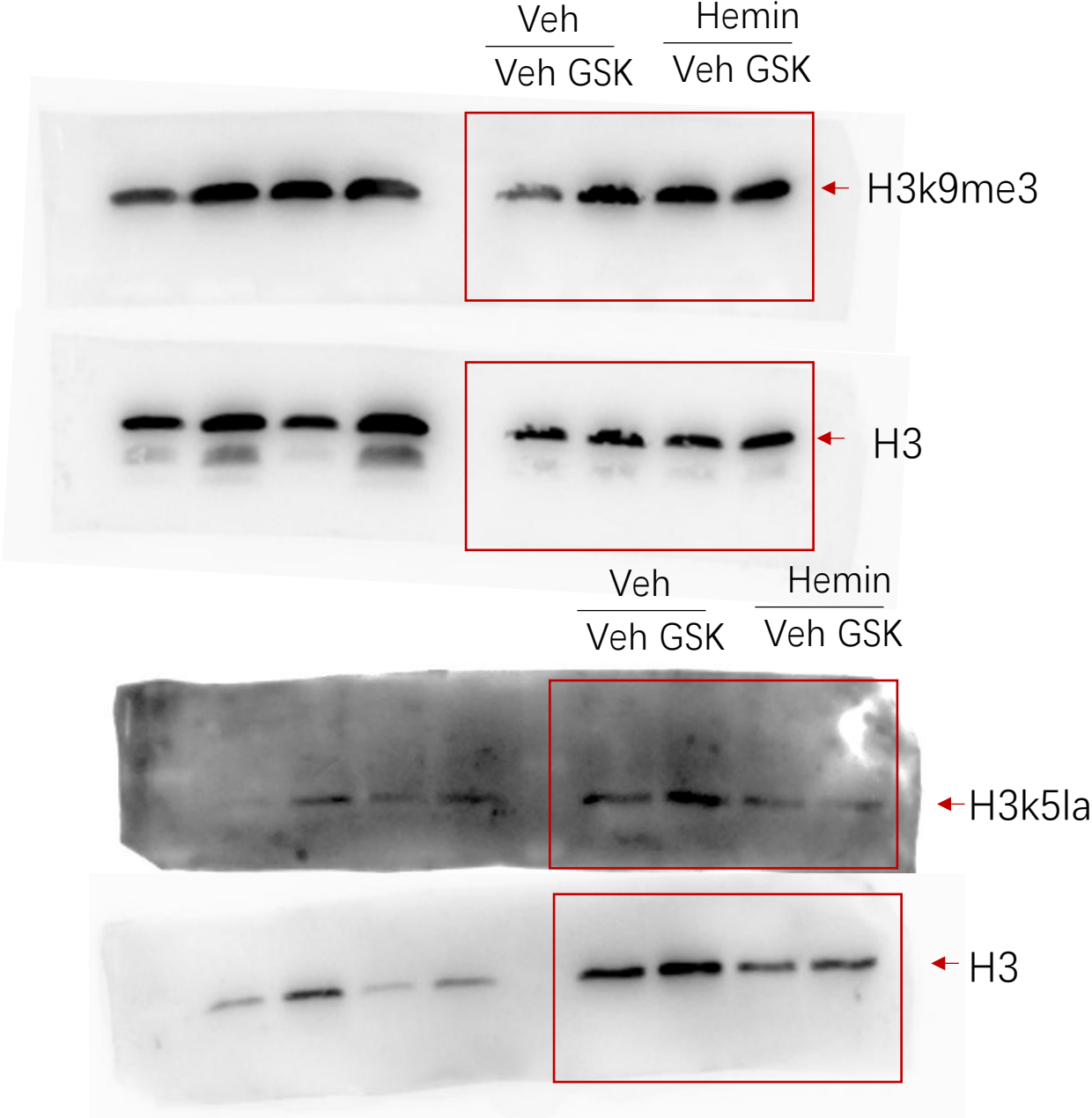

**Fig.3A**

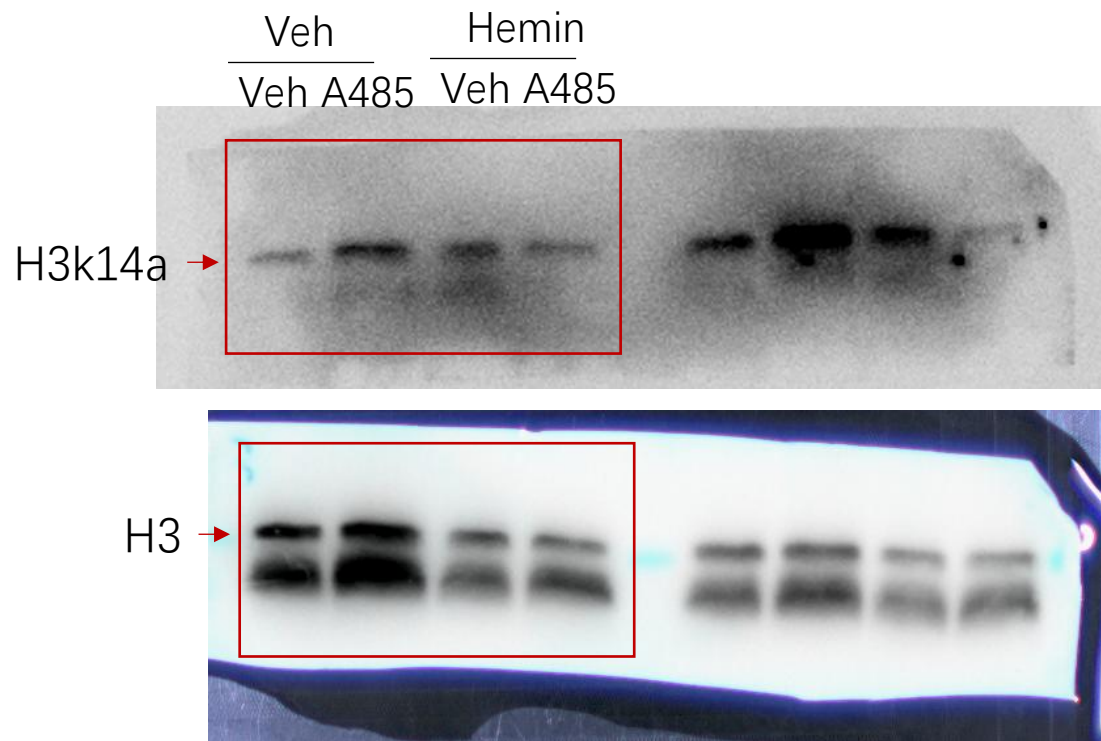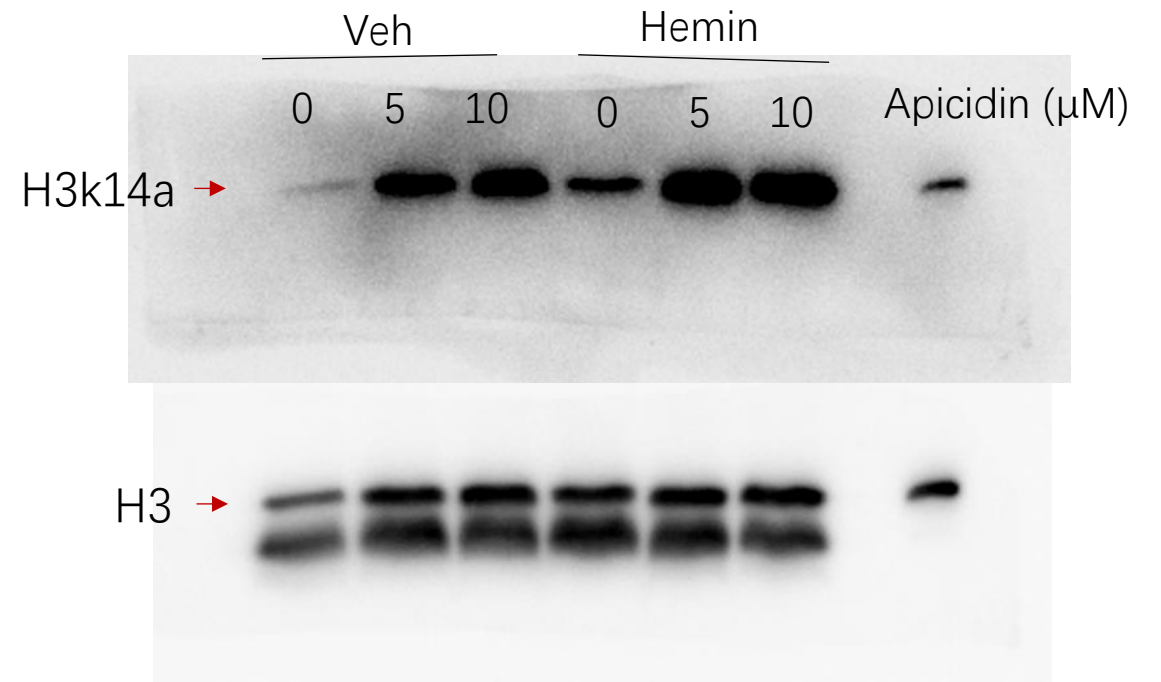

Fig.5E

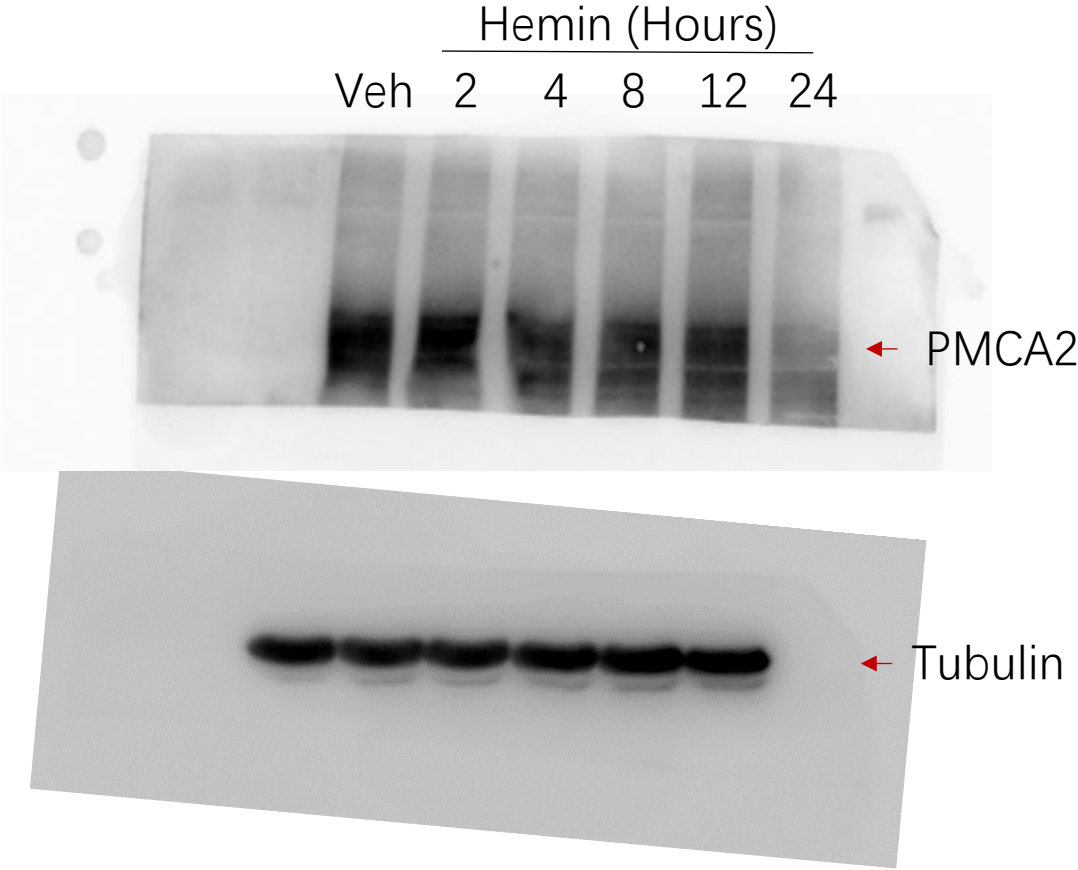

Fig.5J

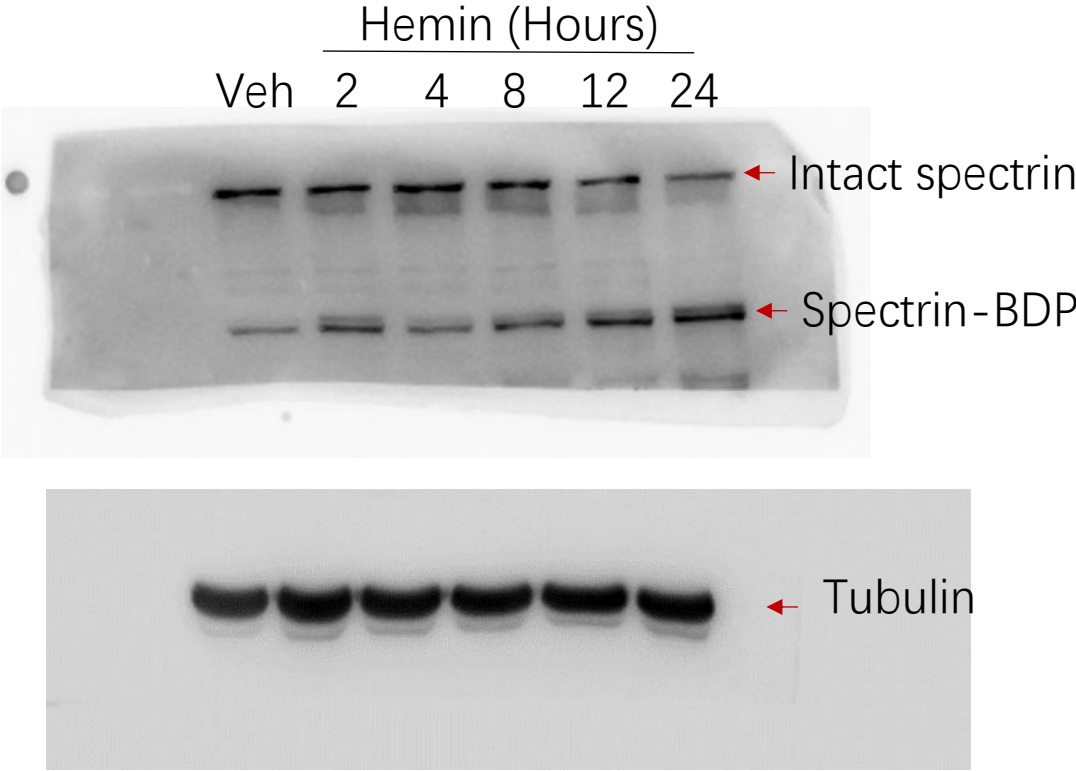

Fig.6C

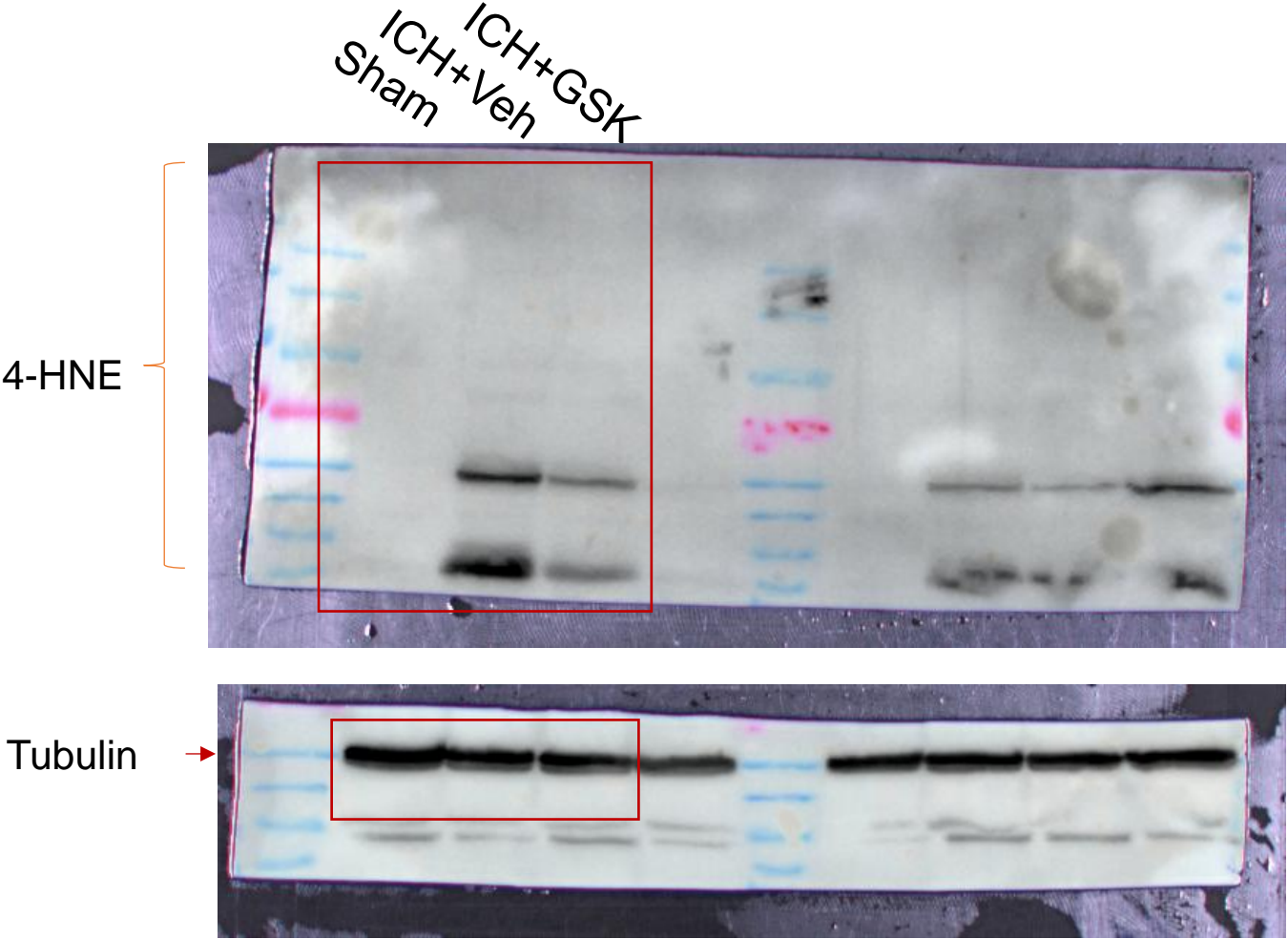

**Fig.S2A**

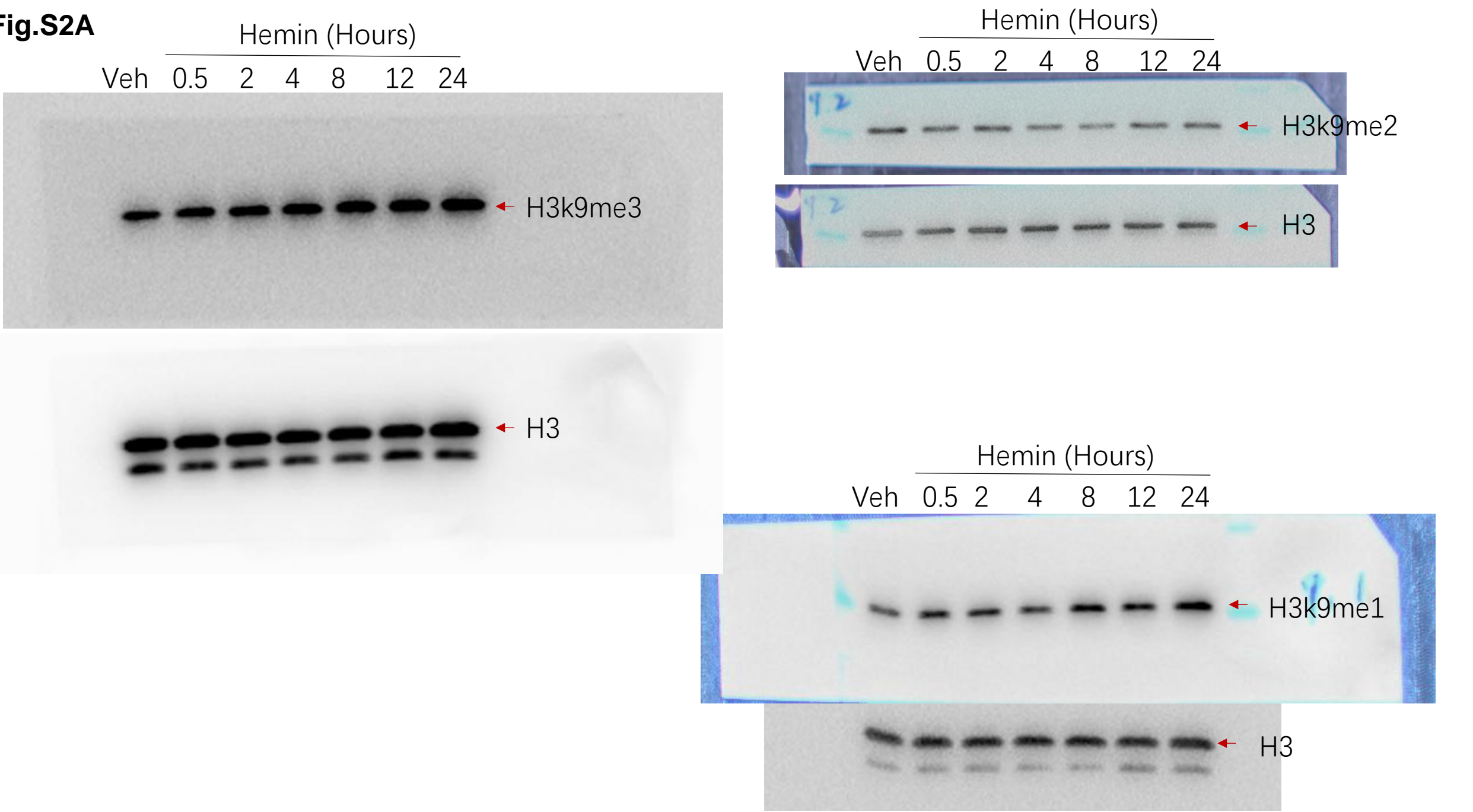

**Fig.S2A**

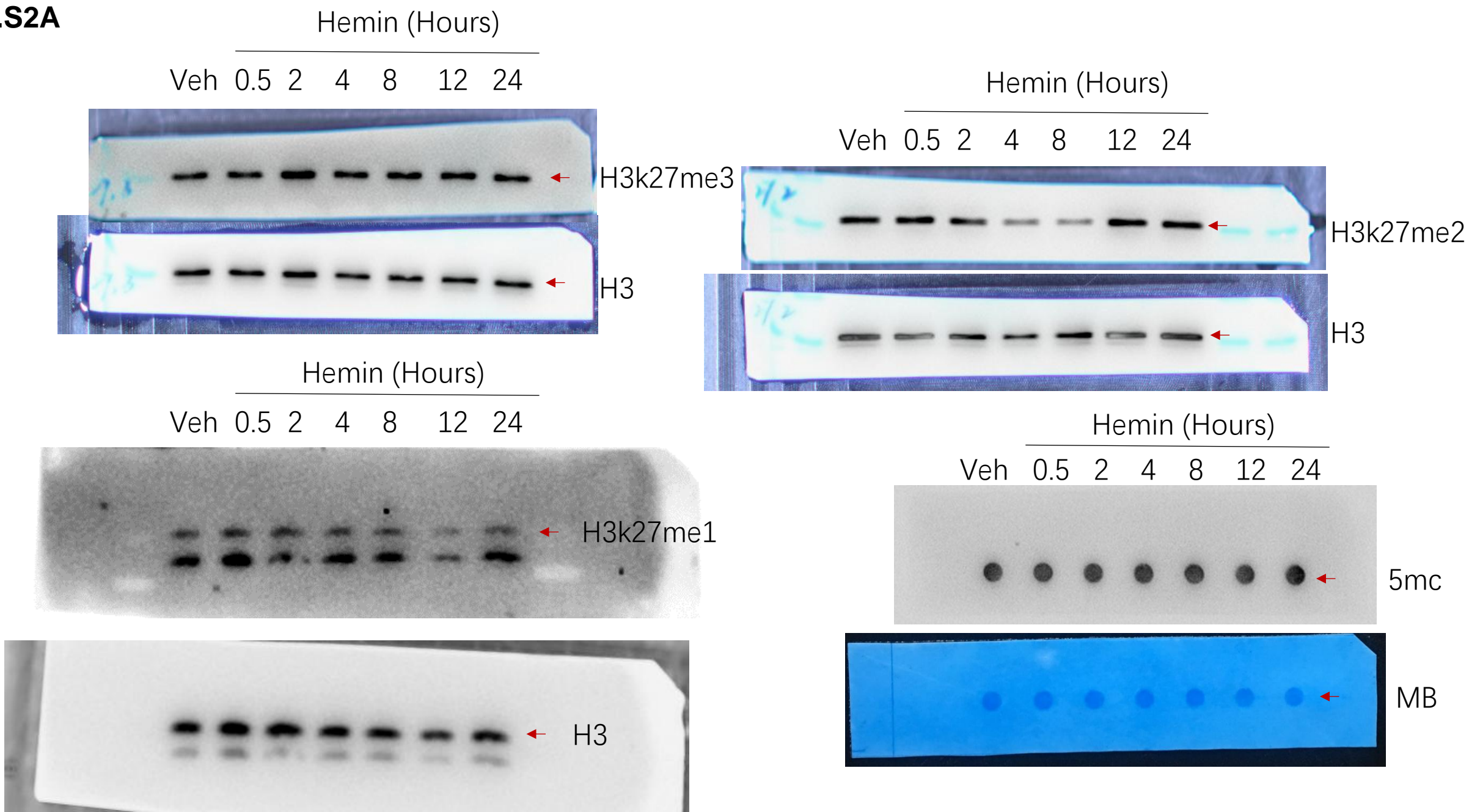

**Fig.S2B**

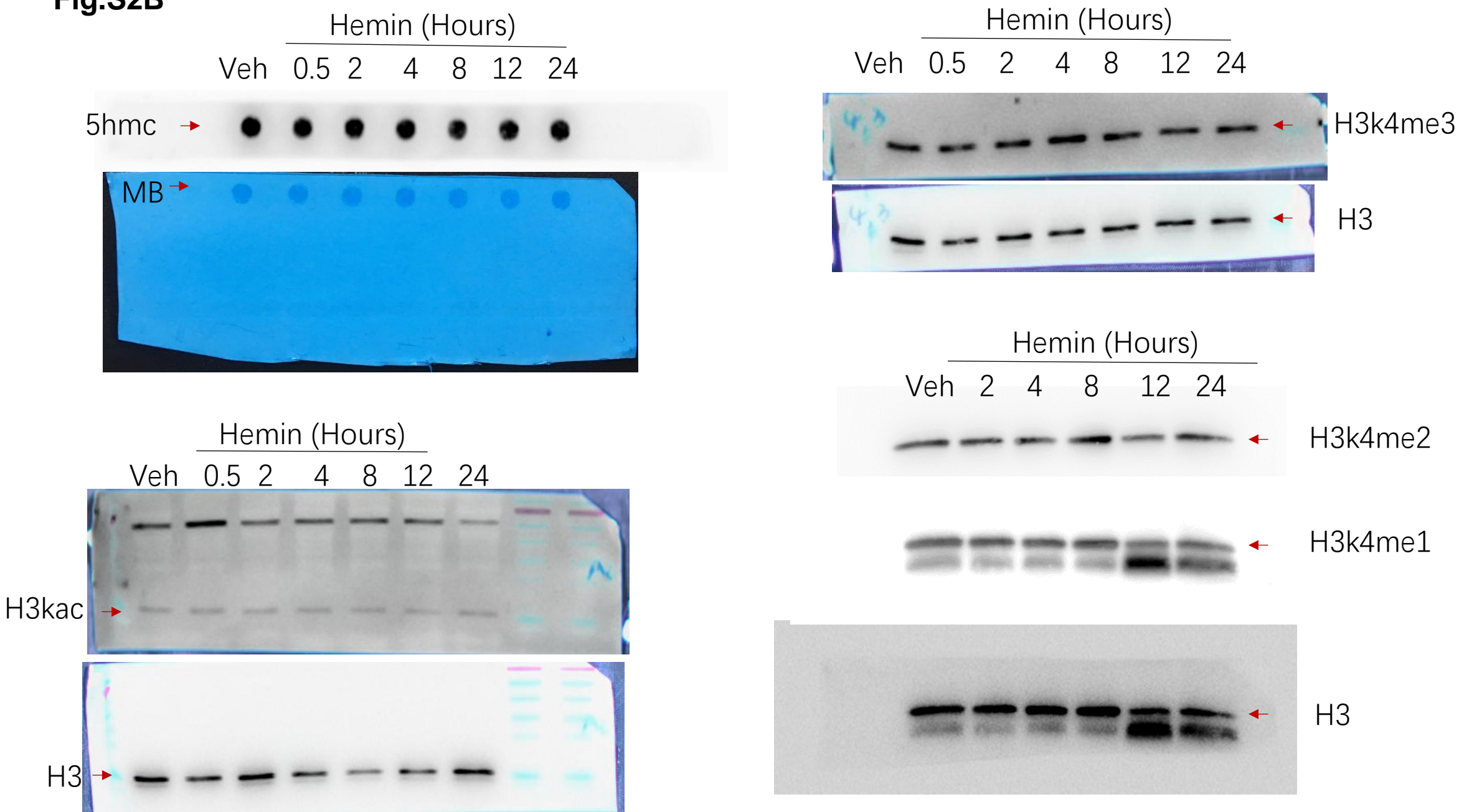

**Fig.S3D**

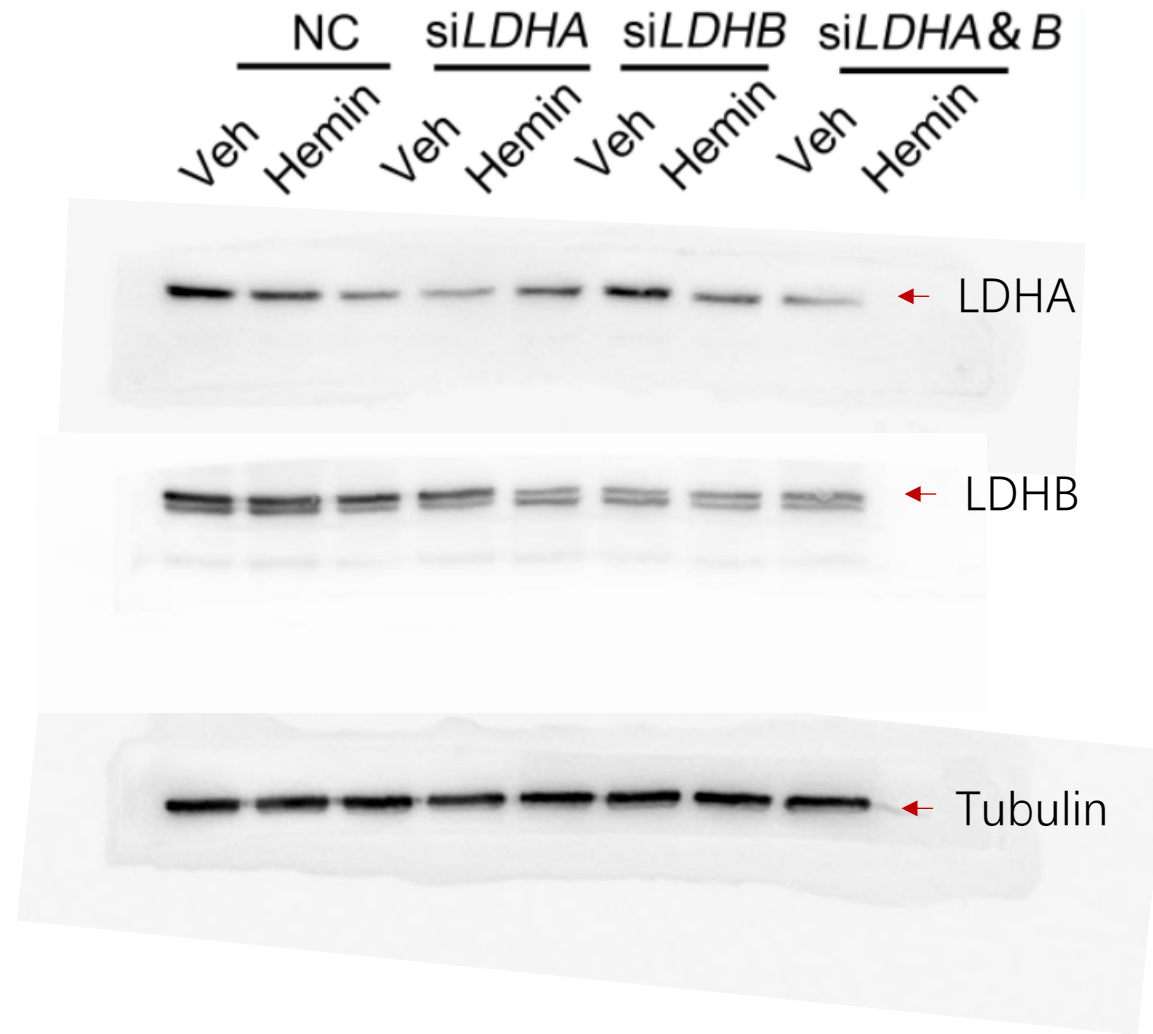

**Fig.S3F**

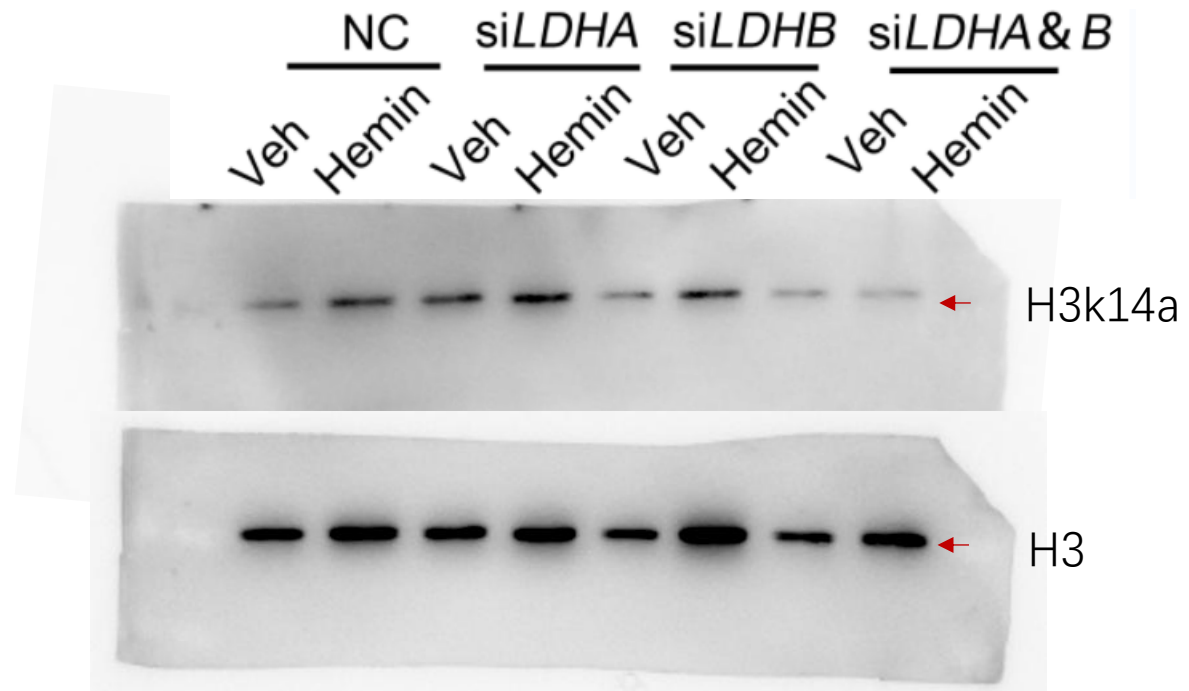

Supplement: Supplementary file 2 — original western blots [file 41419_2025_7874_MOESM2_ESM.pdf]
